# Efficacy and safety of non-invasive brain stimulation in combination with antidepressants in adolescents with depression: a systematic review and meta-analysis

**DOI:** 10.3389/fpsyt.2024.1288338

**Published:** 2024-02-15

**Authors:** Yaoyao Li, Xiaoyan Liu

**Affiliations:** Department of Psychiatry, Affiliated Mental Health Center & Hangzhou Seventh People’s Hospital, Zhejiang University School of Medicine, Hangzhou, Zhejiang, China

**Keywords:** non-invasive brain stimulation, antidepressants, depression, children, meta-analysis

## Abstract

**Objective:**

Non-invasive brain stimulation (NIBS) is beneficial to adult patients with depression, but its safety and efficacy in combination with antidepressants in children and adolescents with depression are not clear. We conducted a preliminary meta-analysis to objectively evaluate its clinical effect and provide information for future research and clinical practice.

**Methods:**

PubMed, Cochrane Library, Embase, and Web of Science were searched systematically to find clinical trials published in English before April 11, 2023. Stata software was used for meta-analysis, and random or fixed effect models were used to combine effect sizes.

**Results:**

Nine studies were eligible and included (n = 393). No articles about children were included in the analysis. The results showed that the remission rate was 40% (95% confidence interval [CI]: 13% to 71%). The scores of Children’s Depression Rating Scale (CRDS) and Hamilton’s depression scale (HAMD) significantly decreased compared to baseline value (MD = -27.04, 95% CI: -30.95, -23.12 and MD = -12.78, 95% CI: -19.55 to -6.01). In addition, the incidence of all adverse events was 13% (95% CI: 5%, 23%), and all were minor pain-related events.

**Conclusion:**

The combination of NIBS and antidepressants has been shown to notably alleviate depressive symptoms in adolescents, offering a considerable level of safety. This therapeutic synergy is particularly effective in patients with major depressive disorder, where repetitive transcranial magnetic stimulation augmented with antidepressants can enhance the amelioration of depressive symptoms.

**Systematic review registration:**

https://www.crd.york.ac.uk/prospero/display_record.php?ID=CRD42023442215, PROSPERO CRD42023442215.

## 1 Introduction

Depression is one of the most common mental health conditions in the world, affecting about 4.4% of the global population (1). Data from a national epidemiological survey showed that the prevalence of depression among adolescents ranged from 11% to 15%, and about 20% of adolescents had major depression at 18 years of age (2–4). Considering the high incidence and potential harm of this disease, World Health Organization ranks major depression as the third leading cause of the global disease burden and predicts that the disease will rank first by 2030 (5). According to reports, depression can have a detrimental impact on the functioning and mental health of children and adolescents, leading to self-harm and suicidal behaviors (6–8). A report in 2021 pointed out that during 1950-2019, deadly self-mutilation among 10 to 24-year-olds accounted for 8.2% of deaths in that age group (9). A matched case-control study conducted by Sumner et al., using real-world network data, found that after adjusting for other factors, the rate of severe self-harm or suicidal behavior among depressed adolescents was 1.82 times higher than that of non-depressed individuals (95% confidence interval [CI]:1.63-2.03) (10).

In order to reduce the harm of depression in young people, it is important to choose effective and safe treatment, besides taking preventive strategies to reduce the incidence of depression. The first-line therapy for depression is psychotherapy and medication. Psychotherapy mainly includes cognitive behavior therapy, interpersonal relationship therapy, acceptance, and commitment therapy (11–14). The medications mainly encompass SSRIs, selective Norepinephrine reuptake inhibitors, and Norepinephrine reuptake inhibitors (15, 16). People who do not respond to antidepressant medications may benefit from switching or adding other medications. Additionally, combination therapy (psychological therapy plus medication) may be more effective than monotherapy (17).

Despite the availability of various medications and psychotherapy approaches, there is still a lack of sufficient clinical response in over 40% of adolescents with major depression to various medication, psychotherapy, or combination of both. Only approximately 60% of adolescent depression patients exhibit adequate clinical response to selective SSRIs (18). A study on the treatment of adolescent depression found that after 12 weeks of treatment, only 23% of patients achieved complete symptom remission (medication combined with cognitive therapy: 37%; cognitive behavioral therapy: 16%; placebo: 17%) (19). In terms of current treatment methods, there is an approximate recurrence rate of 42% to 70% (20, 21). In addition, the safety of therapy is also a concern. Emslie et al. found that at least 2% of patients receiving drug plus psychotherapy experienced sedation, insomnia, vomiting, and epigastric pain, twice as often as those receiving a placebo (22). A review of previous studies by Hammad et al. showed a significant increase in suicidal thoughts in adolescent patients treated with selective SSRIs (23). Therefore, given the current controversies surrounding clinical response, relapse, and safety of existing treatment options, alternative supplementary treatment strategies are being explored.

NIBS, as an alternative treatment for depression, is gaining increasing attention. It regulates the cortical excitability and neural activity of the brain to achieve emotional intervention. Common NIBS techniques include transcranial direct-current stimulation (tDCS), transcranial magnetic stimulation (TMS), and electroconvulsive therapy (ECT) (24). Numerous studies have evaluated the therapeutic benefits of the NIBS model for a wide range of adult patients with mental disorders, such as schizophrenia, anxiety, and depression (25–27). However, most of the research was done in adult populations. Some studies have revealed that NIBS may play a positive role in treating serious mental disorders in children, such as autism spectrum disorder, schizophrenia, attention deficit hyperactivity disorder, and dyslexia (28, 29). Furthermore, NIBS can promote the rehabilitation of dyskinesia after brain injury in children by modulating cortical excitability and enhancing motor learning (30). A prospective trial combining 3.5 million stimuli sessions identified good safety and tolerability of TMS or tDCS in children (31). Although NIBS has achieved good results in the treatment of depression in children and adolescents, who even respond better to rTMS than adults (32), the safety and efficacy of NIBS combined with antidepressants in the treatment of childhood and adolescent depression are still unclear due to the lack of meta-analysis aggregate data. Thus, the aim of this single-arm meta-analysis is to determine whether the combination of antidepressant medication and NIBS has a positive impact on children and adolescent patients with depression, particularly in terms of clinically meaningful improvement, and to evaluate the safety of this treatment. The summarized preliminary evidence may offer more choices for future research and clinical practice.

## 2 Methods

The study was conducted according to the Preferred Reporting Items for Systematic Reviews and Meta-Analyses (PRISMA) (33). The study protocol was registered on PROSPERO, the international prospective register of systematic reviews (registration No CRD42023442215).

### 2.1 Database search

The retrieval strategy of this study was the combination of key words and free words. PubMed, Cochrane Library, Web of Science, and Embase were searched from database inception to April 11, 2023 and language was limited to English. In addition, to reduce the risk of omission, cross-referencing was performed for review articles and meta-analyses. The specific search terms were as follows: (“Depressive Disorder” OR “depression”) AND (“Child” OR “Children” OR “Adolescent” OR “Teens” OR “Youth” OR “Teenager”) AND (“Transcranial Magnetic Stimulation” OR “Electroconvulsive Therapy” OR “Magnetic Field Therapy” OR “Transcranial Direct Current Stimulation” AND (“Antidepressive Agents” OR “Antidepressant”). **Supplementary Table S1** shows the specific search strategy.

### 2.2 Selection criteria

Studies meeting the following inclusion criteria were included in this meta-analysis: 1) Participants: children and adolescents diagnosed with depression (6-25 years) (34). The diagnostic criteria for depression followed the Diagnostic and Statistical Manual of Mental Disorders (third edition, fourth edition, or text revision of the fifth edition) or the International Classification of Diseases (ninth or tenth edition), with no restrictions on disease severity. If explicit diagnostic criteria are not provided in the study, it is necessary to make inferences about patient’s depression based on existing information in the research (e.g., selecting patients currently undergoing depression treatment).

2) Intervention: the patients were treated with conventional antidepressants combined with NIBS, including TMS (including θ burst stimulation), tDCS, ECT and other non-invasive stimulation.

3) Outcome: the studies reported one or more of the following outcomes, including changes in depression scores compared to baseline, relief of depressive symptoms, or rate of adverse events. There is currently no unified standard for outcome measurement (35). Based on the current research status, we have developed the following criteria. Depression score measures should include at least one of the following: Childhood Depression Rating Scale (CDRS), revised CDRS (CDRS-R), Hamilton Depression Scale (HAMD), Original Beck Depression Inventory (BDI-I), or Beck Depression Inventory-II (BDI-II). The criteria for remission should meet one of the following: HAMD ≤ 7, CDRS-R ≤ 28, BDI < 10, or clinical judgment based on follow-up. The criteria for other efficacy outcomes are as follows: Early improvement: a 20%-25% reduction in HAMD score; Response: at least a 50% reduction in HAMD score, CDRS-R score ≤ 40, at least a 30% reduction in CDRS-R score, at least a 50% reduction in BDI-I score, or at least a 30% reduction in BDI-II score; Recovery: maintaining the remission state for more than 2 months. The same outcome was reported in ≥ 2 studies.

4) Type of study: clinical intervention trial (single arm trial, randomized controlled trial (RCT) or non-RCT (with external parallel control group, but non-randomized allocation).

Exclusion criteria were as follows: animal experiments, cellular studies, reviews, guidelines, abstracts, conference papers, meta-analyses, case reports and letters were excluded, for these types of publications often lack important quantitative information. Two reviewers independently identified eligible articles based on inclusion and exclusion criteria.

### 2.3 Data extraction and quality assessment

Information on all included studies was independently extracted by two reviewers, and the quality of the studies was assessed. The extracted data were summarized as follows: author, year of publication, type of study, country, sample size, sex, age, intervention mode, site of stimulation, diagnostic criteria, duration, and outcome index. Any disagreement was discussed with a third reviewer.

MINORS (Methodological index for non-randomized studies) (36) was used for quality evaluation of single-arm and non-RCTs. MINORS include 12 items: (1) clear study objectives; (2) consistency of inclusion of patients; (3) collected expected data; (4) end-point indicators that appropriately reflected study objectives; (5) objectivity of end-point indicators evaluation; (6) adequacy of follow-up; (7) the rate of loss to follow-up was less than 5%; (8) the sample size was estimated; (9) the selection of the control group was appropriate; (10) the control group was synchronized; (11) the baseline was comparable between the groups; and (12) the statistical analysis was appropriate. The scoring criteria were: unreported (0 points), reported but insufficient (1 point), reported and adequate (2 points), with a maximum of 24 points. The first eight items were used for single-arm, and all 12 items were used for non-RCTs.

Cochrane ROB2 Tool was used to assess the risk of bias in randomized controlled trials (RCT). The tool included the following five evaluation domains: bias during randomization, bias away from established interventions, bias for missing outcome data, bias for outcome measures, and bias for selective reporting of outcomes. The risk of bias in each domain would be classified as “Low”, “Some concerns” or “High”.

### 2.4 Statistical analysis

In this study, the change in depression scores compared to baseline and remission rate were used as the primary outcome measures, while adverse events and other efficacy outcomes were used as the secondary outcome measures for the quantitative analysis.

All data were analyzed with Stata 16.0 software (StataCorp LP, College Station, TX, USA). Mean difference (MD) was used for continuous variables, and rate was used for dichotomous variables as effect size for pooled results. Meanwhile, 95% CI were reported for all data. For all outcomes, the more conservative random-effects model was used for the initial summary of results. Study heterogeneity was assessed using Cochran’s *I^2^* index and Q test. P < 0.1 represented a statistically significant difference. If there was significant heterogeneity (*I^2^* >50%, P<0.1), the random-effects model was used for subsequent analysis, otherwise, the fixed-effects model was used. When necessary, we also investigated heterogeneity by subgroup analysis and regression analysis for primary outcomes (≥5 studies). To compare the difference in pooled estimates for an outcome measure between different subgroups, we selected the random-effects model in R software (version 4.3.1) and Metafor software to perform regression analysis to obtain the P value of the difference comparison (37, 38). In addition, a sensitivity analysis was conducted to access the effect of a single study on the pooled effects by omitting one study in turn each time (39). Finally, funnel plots were used to assess reporting bias, and Begg’s or Egger’s tests were used to identify potential publication bias (≥10 studies). If there was significant publication bias, the trim-and-fill method was used to measure the impact of publication bias on the results.

## 3 Results

### 3.1 Study selection

**Figure 1:**
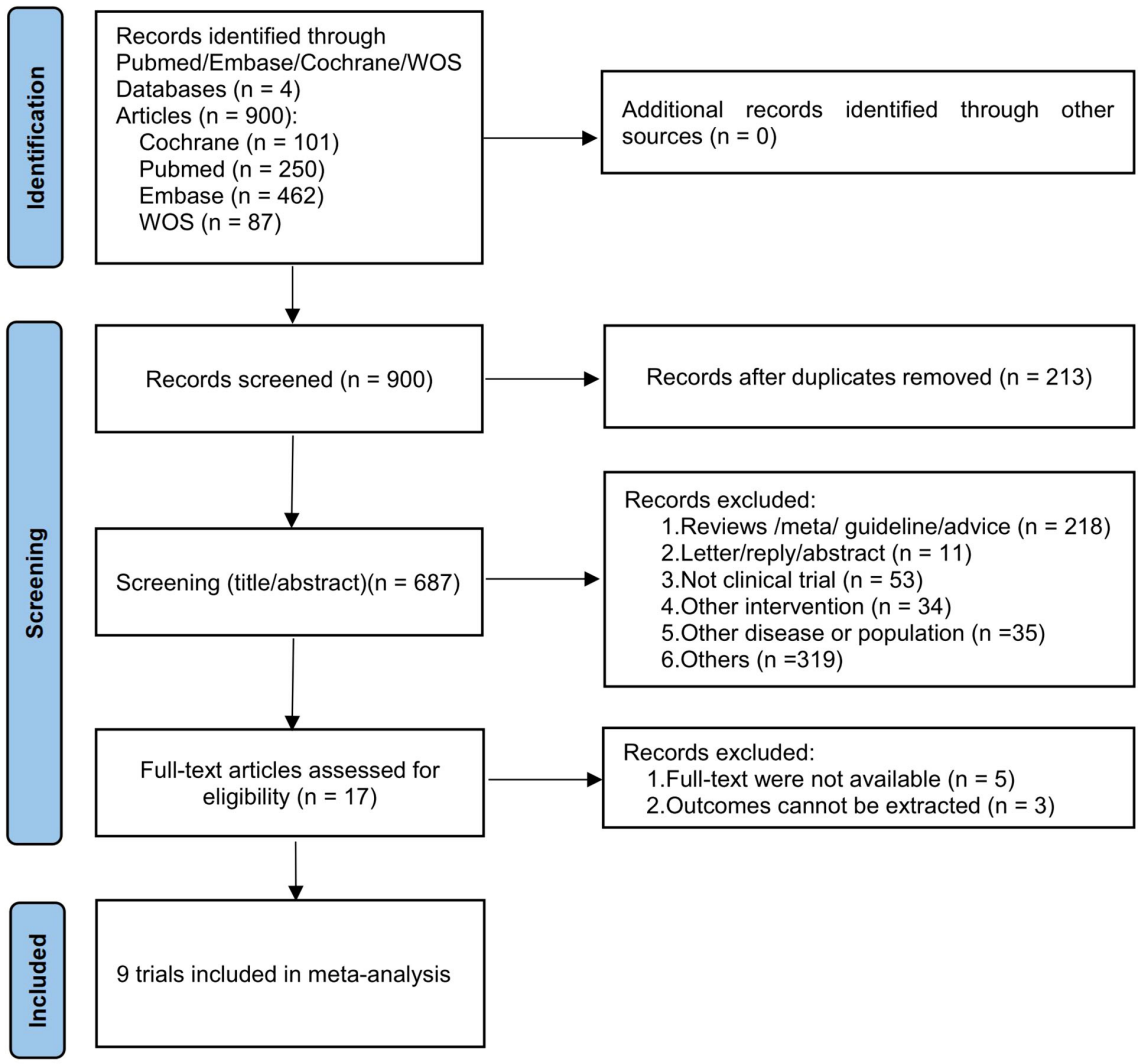
The PRISMA flowchart of the literature search and selection.

A total of 900 records were found in the initial search of four databases, of which 213 were excluded due to duplication. After evaluating the titles and abstracts of 687 records, we selected 17 reports for full-text retrieval and further evaluated their eligibility. After excluding five articles that did not have full texts and three articles that reported irrelevant outcome measures, we finally included nine studies for subsequent meta-analyses (32, 40–47). The process of literature screening is shown in **Figure 1**.

### 3.2 Characteristics of the included studies

The analysis captured data on 393 patients aged between 12 to 25 years: 12-18 years (comprising 8 studies) and 17-25 years (represented by 1 study). A significant majority of the participants, 74.3%, were female. No articles that met the criteria in children were included in the analysis. Within the 9 studies analyzed, 6 targeted patients diagnosed with major depression, 1 investigated individuals with treatment-resistant depression, and 2 did not specify the severity of the depressive symptoms in their patient cohorts. These 9 studies were conducted in a total of 5 countries, including China (3), United States (3), Australia (1), Israel (1), and India (1). One study was RCT, one study was non-RCT, and seven were single-arm clinical trials that compared the improvement in depression before and after self-treatment. In terms of intervention mode, one study chose electro-shock therapy combined with fluoxetine, and the other eight studies chose transcranial magnetic stimulation combined with antidepressants for depression (7 rTMS, 1 TBS). The basic characteristics of the included studies are detailed in **Table 1**.

**Table 1:** Characteristics of the included studies.

First Author	Publication Year	Study Design	Country	Sample size (Male/Female)	Age, years	Intervention (Frequency & Intensity & electricity)	Contol group	Stimulation site	Diagnostic Criteria	Duration, weeks	Level of depression
Bloch et al.	2008	Single-arm	Israel	9(2/7)	17.3 (16-18)	rTMS+antidepressant; 10hz; 80%	NA	L-DLPFC	DSM-IV	2	MDD
Cai et al.	2023	Non-RCT	China	160(23/137)	13-18	ECT+ fluoxetine; 10-70hz; 0.9A	Fluoxetine	L-DLPFC	DSM-V	2	MDD
Chen et al.	2022	RCT	China	97(18/79)	12-18	rTMS+sertraline; 10hz; 90%	Sertraline	L-DLPFC	DSM-IV	2	MDD
Croarkin et al.	2018	Single-arm	USA	19(13/6)	16.00 ± 1.29	rTMS+(either a selective serotonin reuptake inhibitor or serotonin and norepinephrine reuptake inhibitor; 10hz; 120%	NA	L-DLPFC	NM	6	TRD
Rosenich et al.	2019	Single-arm	Australia	15(7/8)	20.69 ± 2.55	rTMS+antidepressant; 10hz; 80%	NA	L-DLPFC R-DLPFC	DSM-IV	6	MDD
Shere et al.	2021	Single-arm	India	26	15.8 ± 1.2	TBS+antidepressant; 10hz; 80%	NA	L-DLPFC R-DLPFC	ICD	2	NM
Sonmez et al.	2020	Single-arm	USA	17(5/12)	15.94 ± 1.35	rTMS+antidepressant; 10hz; 120%	NA	L-DLPFC	DSM-IV	6	MDD
Wall et al.	2011	Single-arm	USA	8(7/1)	16.5 ± 1.18	rTMS+SSRI; 10hz; 120%	NA	L-DLPFC	DSM-IV	6-8	MDD
Zhang et al.	2019	Single-arm	China	42(13/42)	14.6 ± 2.0	rTMS+antidepressant; 10hz; 120%	NA	L-DLPFC	DSM-IV	2	Unclear

rTMS, repetitive transcranial magnetic stimulation; L-DLPFC, left dorsolateral prefrontal cortex; R-DLPFC, right dorsolateral prefrontal cortex; SSRI, Selective Serotonin Reuptake Inhibitors; BTL, bilateral temporal lobes; BDI, Beck Depression Inventory; ICD, International Classification of Diseases; DSM, Diagnostic and Statistical Manual of Mental Disorders; NM, not mentioned; NA, not applicable; age (mean ± standard deviation); age (range);RCT, Randomized Controlled Trials; MDD, major depressive disorder; TRD, treatment-resistant depression

### 3.3 Quality assessment

**Figure 2:**
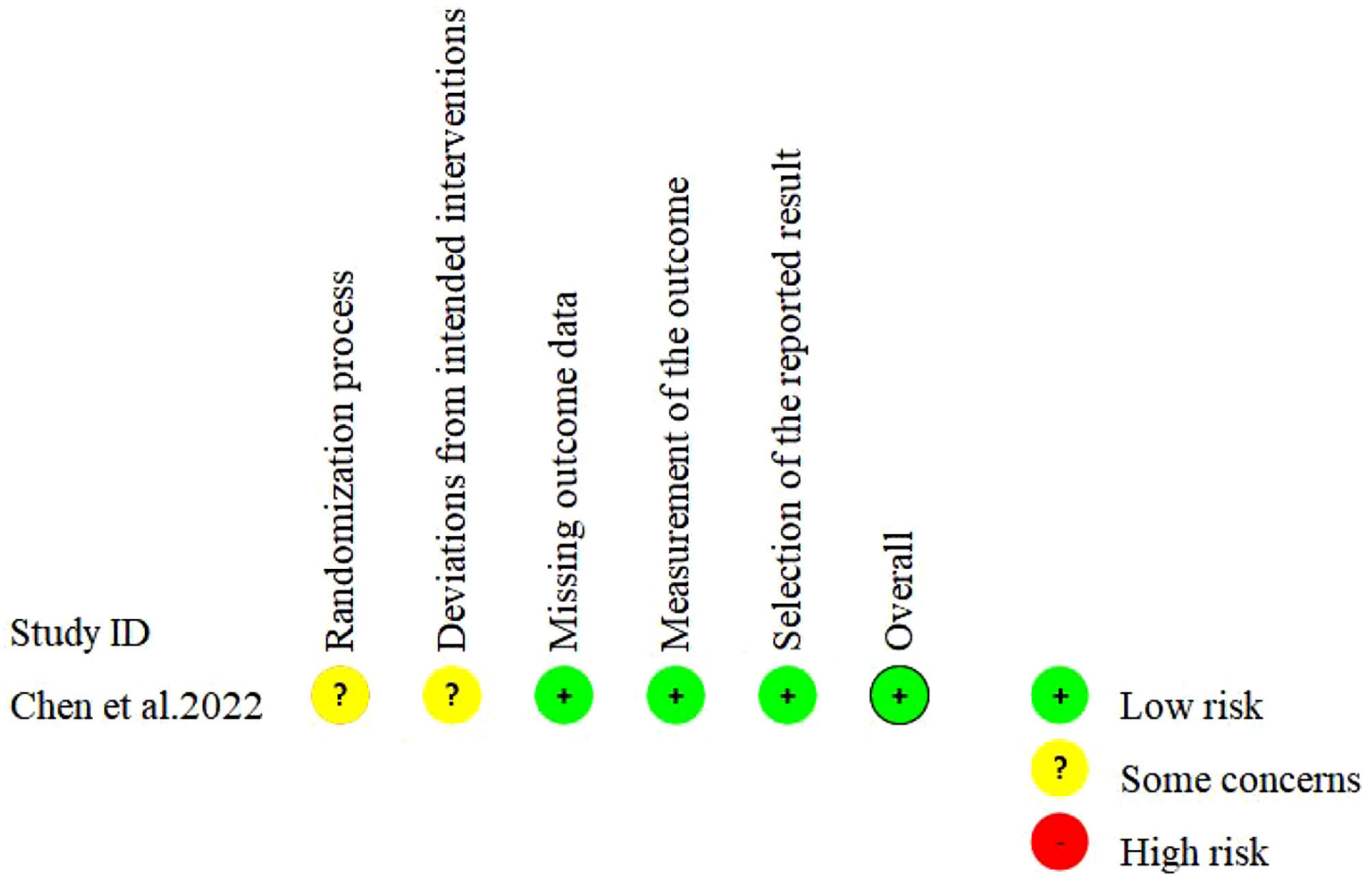
Assessment of risk of bias in the included studies (RCTs).

Seven studies were single-arm trials and one was non-RCT. The quality evaluation of MINORS showed that the included studies reported no sufficient information on the objectivity of the evaluation of end-point indicators, whether the sample size was estimated prospectively and whether adequate follow-up time was set. All single-arm trials had a total score of 13-14 (moderate quality) and the non-RCT had a total score of 22 (high quality) (**Table 2**). One study was RCT and the Cochrane risk of bias assessment showed an overall low risk (**Figure 2**).

**Table 2:** Quality assessment of included studies (Single-arm trials and non-RCT).

Author	Year	A	B	C	D	E	F	G	H	I	J	K	L	Total
Bloch et al.^*^	2008	2	2	2	2	2	1	2	0	/	/	/	/	13
Croarkin et al.^*^	2018	2	2	2	2	2	1	2	0	/	/	/	/	13
Rosenich er al.^*^	2019	2	2	2	2	1	2	2	1	/		/	/	14
Shere et al.^*^	2021	2	2	2	2	2	1	2	0	/		/	/	13
Sonmez et al.^*^	2020	2	2	2	2	2	2	2	0	/		/	/	14
Wall et al.^*^	2011	2	2	2	2	2	2	2	0	/		/	/	14
Zhang et al.^*^	2019	2	2	2	2	1	2	2	1	/	/	/	/	14
Cai et al.^#^	2023	2	2	2	2	2	2	2	0	2	2	2	2	22

*, Single-arm trials; #, non-RCT; /, not applicable. Numbers A-H in heading signified: A, clearly stated study objectives; B, Inclusion of consecutive patients; C, Prospective collection of data; D, Endpoints appropriate to the aim of the study; E, Unbiased assessment of the study endpoint; F, Follow-up period appropriate to the aim of the study; G, Loss to follow up less than 5%; H, Prospective calculation of the study size; I, A control group having the gold standard intervention; J, Contemporary groups; K, Baseline equivalence of groups; L, Statistical analyses adapted to the study design.

### 3.4 Results of meta-analysis

Primary outcomes measures were post-treatment remission rates and change from baseline in depression scores. Secondary outcomes were post-treatment adverse events and other efficacy outcomes.

#### 3.4.1 Remission rate

**Figure 3:**
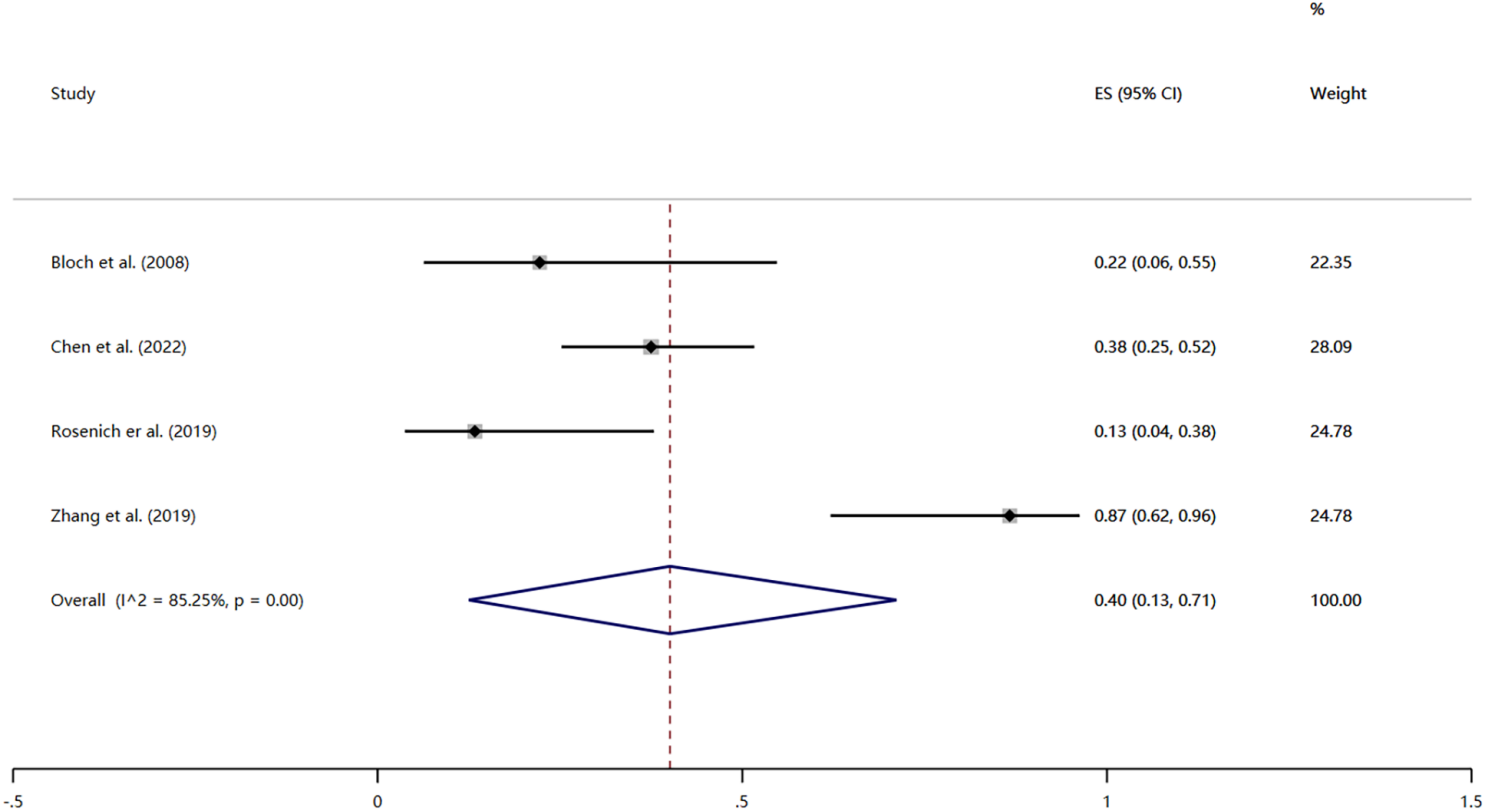
Forest plot of remission rate.

Four studies reported the remission rates after treatment (32, 40, 42, 44). Remission was defined as HAMD score ≤7 or judged by regular clinical follow-up. The heterogeneity test revealed significant heterogeneity among the included studies (*I^2^* = 85.25%, P < 0.01); thus the results were combined using a random effects model. The remission rate was 40% (95% CI: 13% to 71%; P < 0.01) (**Figure 3**).

#### 3.4.2 CRDS score

**Figure 4:**
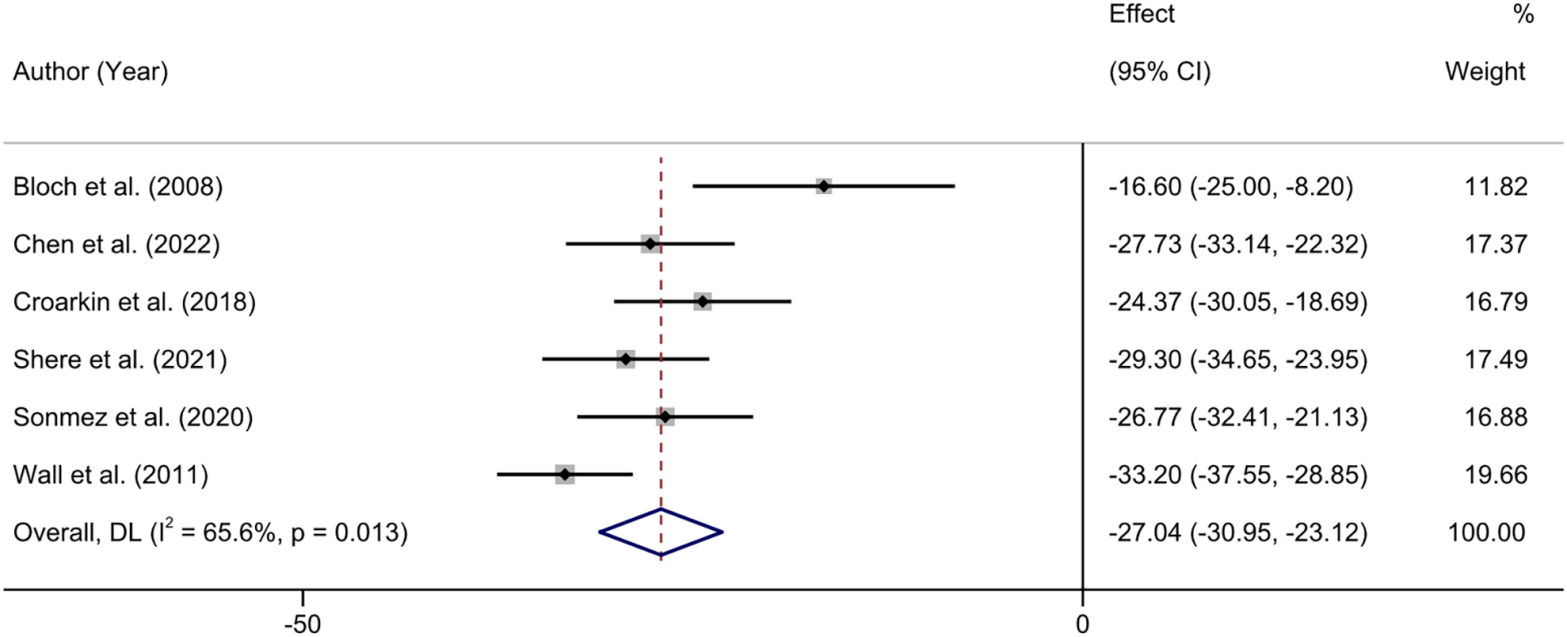
Forest plot of CRDS score.

Six studies reported the changes in CRDS scores before and after treatment (40, 42, 43, 45–47). Heterogeneity test showed that there was moderate heterogeneity among the included studies (*I^2^* = 65.6%, P = 0.013), so we used the random effects model to combine the results. The meta-analysis showed that NIBS combined with antidepressants significantly reduced patients’ CRDS depression scores (MD = -27.04; 95% CI: -30.95, -23.12; P < 0.001) (**Figure 4**).

#### 3.4.3 HAMD score

**Figure 5:**
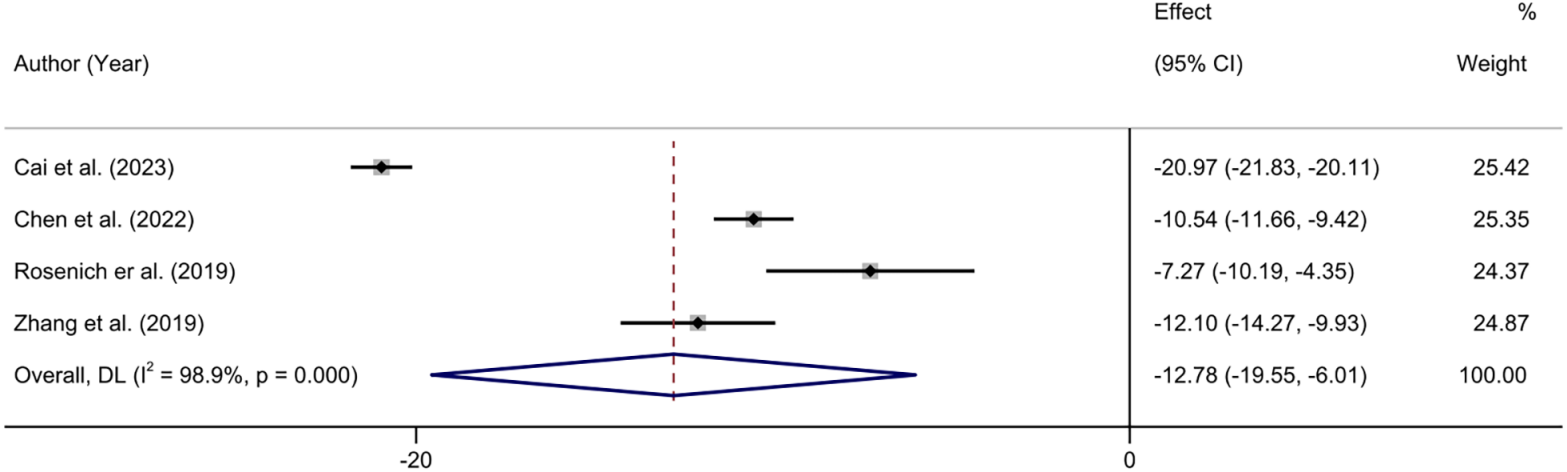
Forest plot of HAMD score.

Four studies reported changes in HAMD scores before and after treatment (32, 41, 42, 44). The heterogeneity test showed that there was significant heterogeneity among the included studies (*I^2^* = 98.9%, P < 0.01). The results showed that the HAMD scores of the patients after treatment were significantly lower than those before treatment (MD = -12.78.95% CI: -19.55 to -6.01; P < 0.001) (**Figure 5**).

#### 3.4.4 Other efficacy outcomes

Early rates of improvement and response were reported in 2 studies and 6 studies, respectively. Summary results are as follows, early improvement rate after treatment was 95% (95% CI: 87% to 99%; P < 0.01), the response rate of 63% (95% CI: 41% to 83%; P < 0.01) (**Supplementary Figures S4**, **S5**).

#### 3.4.5 Rate of adverse events

**Figure 6:**
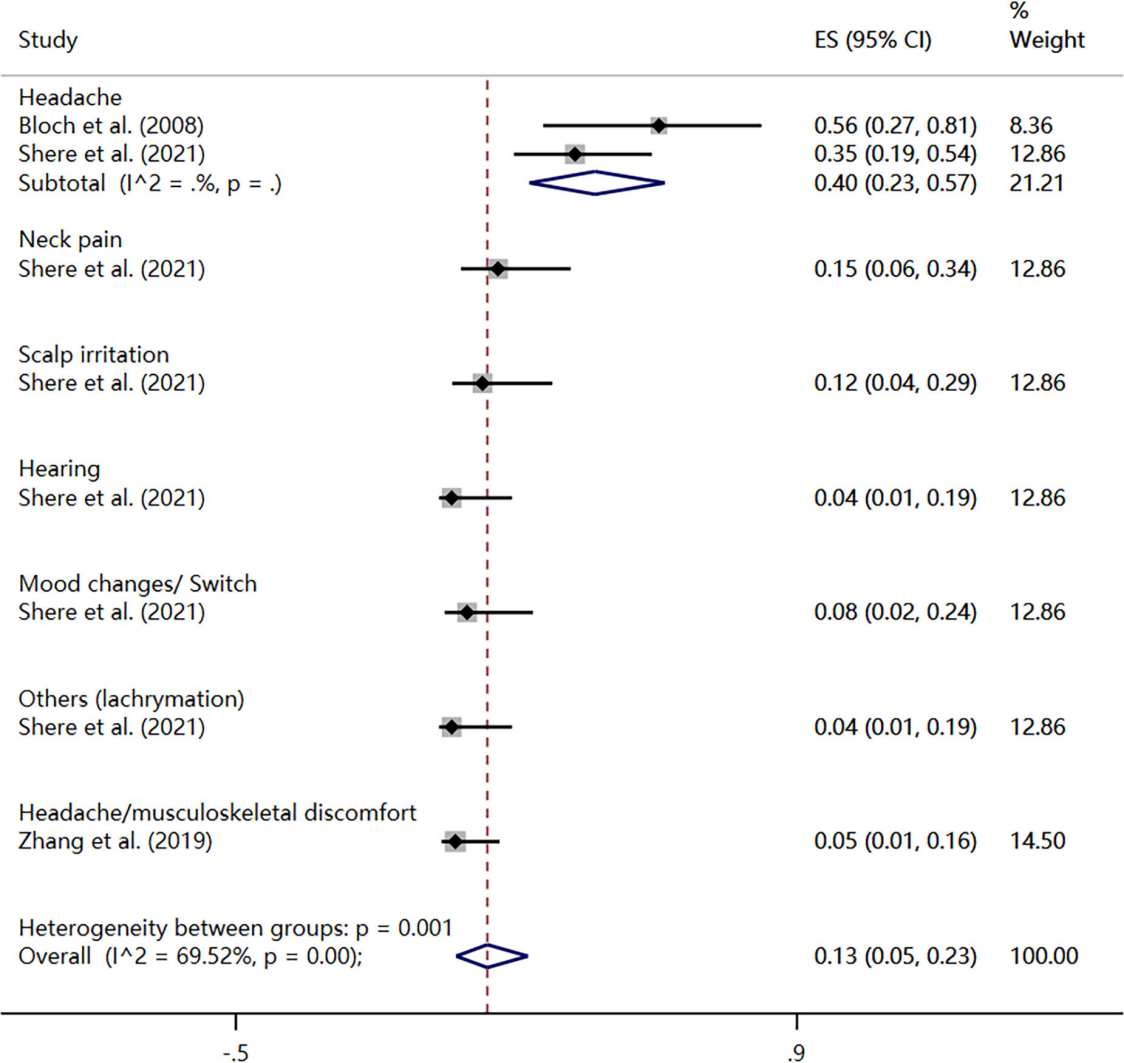
Forest plot of adverse effect rate.

Three studies reported the rate of adverse events during treatment (32, 40, 45), with a total type of 7 events. The heterogeneity test showed that there was significant heterogeneity among the included studies (*I^2^* = 69.52%, P < 0.01), so the results were pooled using a random-effects model. The meta-analysis revealed an overall incidence rate of adverse events at 13% (95% CI: 5%, 23%). Among them, the incidence rate of headache was 40% (95% CI: 23%, 57%); the incidence of neck pain was 15% (95% CI: 0.06%, 0.34%); the incidence of scalp irritation was 12% (95% CI: 4%, 29%); hearing and lachrymation both had an incidence rate of 4% (95% CI: 1%, 19%); mood changes or switch had an incidence rate of 8% (95% CI: 2%, 24%); headache or musculoskeletal discomfort had an incidence rate of 5% (95% CI: 1%, 16%) (**Figure 6**).

#### 3.4.6 Subgroup analysis and regression analysis

Subgroup analyses of CRDS score were performed to explore the source of heterogeneity according to intervention measures, treatment duration and measurement methods, as shown in **Table 3**.

**Table 3:** Subgroup analyses of CRDS score was performed according to intervention measures, duration of treatment, diagnostic scales. *, P<0.05.

Outcomes	Subgroups	Number of studies	ES	95%CI	Heterogeneity (Intra-group)		Heterogeneity (between groups)
					*I^2^*	P	P
CRDS score	Intervention						
	rTMS+Antidepressants	5	-26.42	-31.23, -21.61	71.9%	0.007	0.432
	TBS+Antidepressants	1	-29.30	-34.65, -23.95	—	—	
	Duration of treatment						
	2 weeks	3	-25.31	-31.84, -18.78	69.6%	0.037	0.483
	6-8 weeks	3	-28.37	-33.89, -22.86	70.3%	0.035	
	Measurement methods						
	CDRS	2	-21.13	-28.64, -13.62	55.6%	0.133	0.039^*^
	CDRS-R	4	-29.62	-32.61, -26.63	25.9%	0.256	

*P<0.05.

Subgroup analysis of CRDS score was performed based on treatment duration. The results showed that the CRDS score was MD = -25.31(95% CI: -31.84, -18.78; P < 0.001) at 2 weeks and MD = -28.37(95% CI: -33.88, -22.86; P < 0.001) at 6-8 weeks (**Supplementary Figure S1**). The differences between subgroups were not statistically significant (P=0.472). The differences between subgroups were not statistically significant (P = 0.483). In addition, we also conducted a subgroup analysis according to the intervention, and the CRDS score for RTMS plus antidepressants was MD = -26.42(95% CI: -31.23, -21.61; P < 0.001); The CRDS score for TBS plus antidepressants was MD = -29.30(95% CI: -34.65, -23.95; P < 0.001) (**Supplementary Figure S2**). The choice of additional noninvasive brain stimulation modality, rTMS or TBS, did not significantly affect the combined CRDS score between subgroups (P = 0.601), which may be due to the fact that TBS remains, in principle, one of the modes of TMS implementation. Finally, after subgroup analysis based on CDRS score, there were significant differences between subgroups (P=0.02). The conventional CDRS showed MD = -21.13(95% CI: -28.64, -13.62; P < 0.001) while corrected CDRS showed MD = -29.62(95% CI: -32.61, -26.63; P < 0.001) (**Supplementary Figure S3**). It is possible that the corrected CDRS (CDRS-R) can better capture the improvement of depression. Because of the limited number of studies, no regression analysis for heterogeneity of CRDS scores. In addition, we did not perform subgroup and regression analyses of HAMD and response rates because the numbers within each subgroup were too small.

#### 3.4.7 Sensitivity analysis

**Figure 7:**
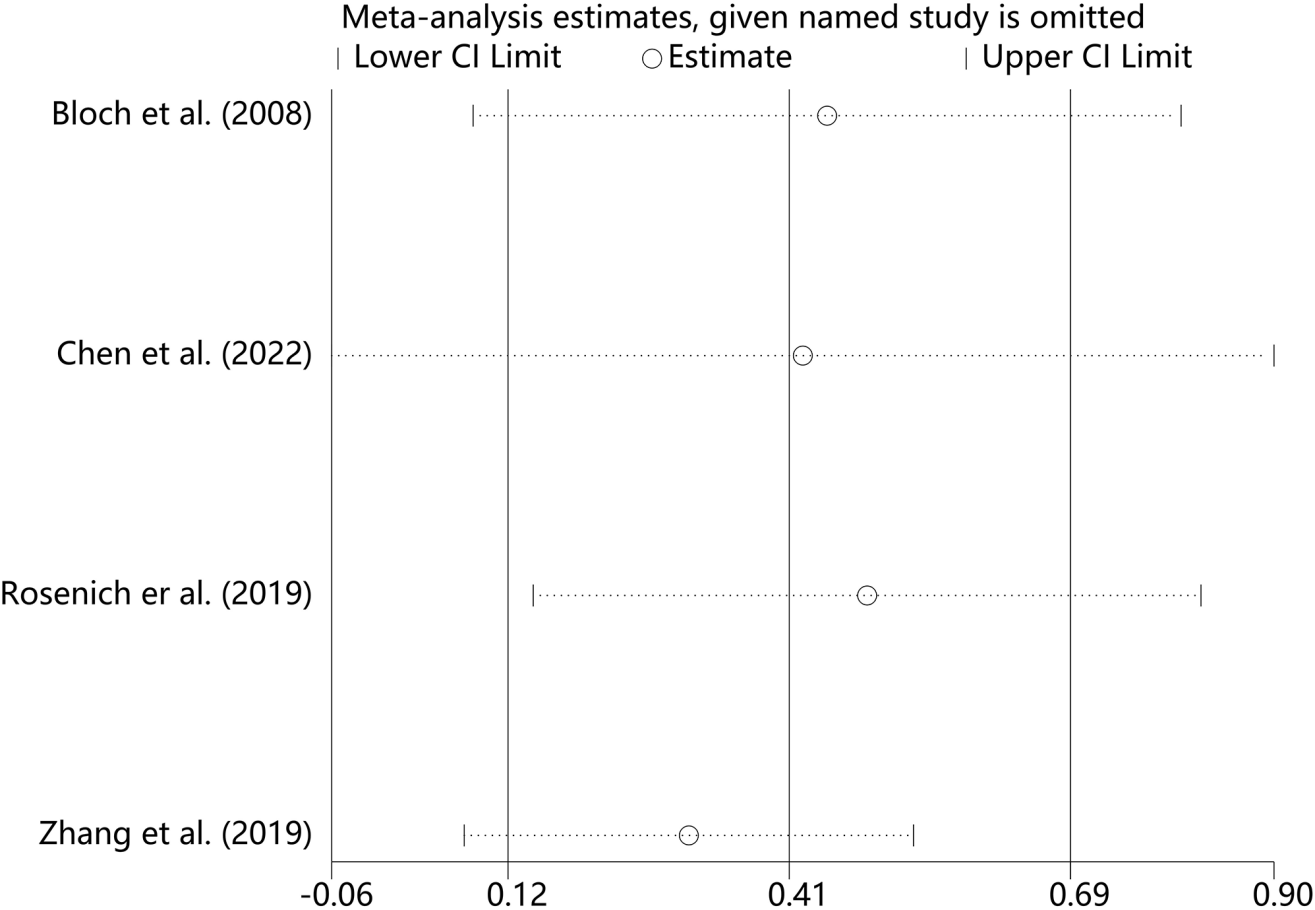
Sensitivity analysis for the remission rate by sequentially excluding each individual trial.

**Figure 8:**
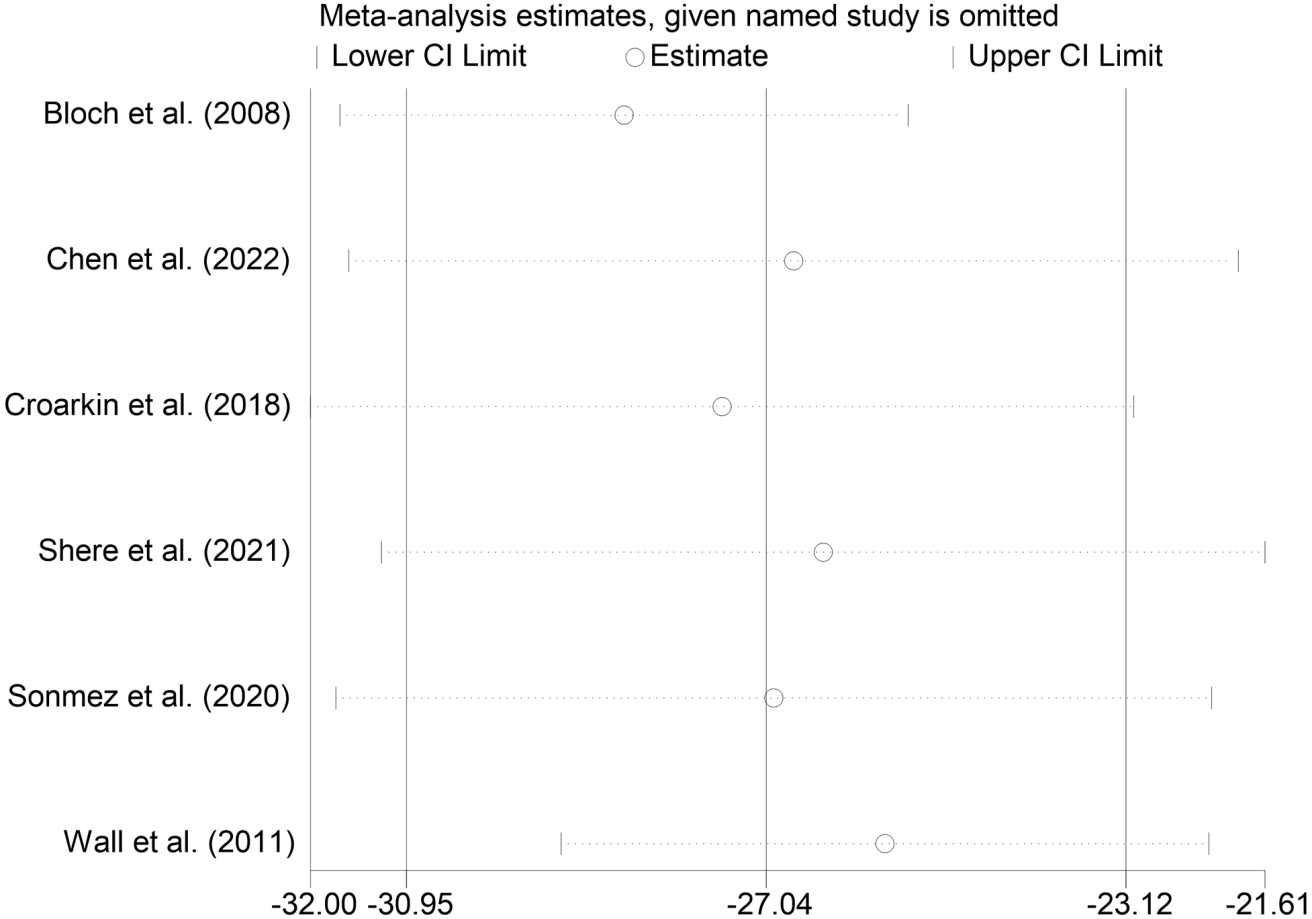
Sensitivity analysis for the CRDS score by sequentially excluding each individual trial.

**Figure 9:**
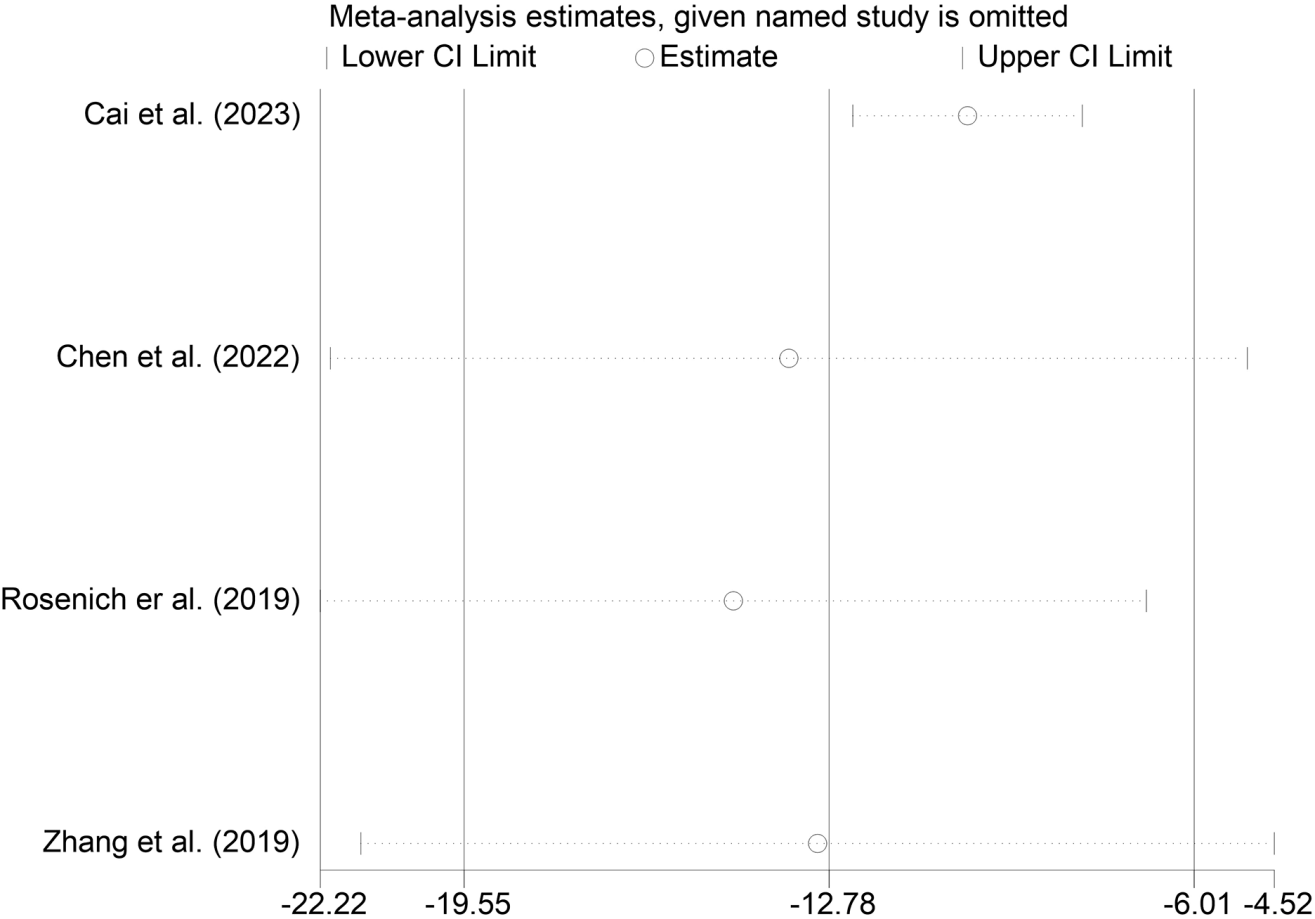
Sensitivity analysis for the HADS score by sequentially excluding each individual trial.

We performed sensitivity analyses on the main outcome indicators to assess the impact of individual studies on the pooled results by excluding them individually. Considering that the age of the subjects included in Rosenich’s study (41) differed markedly from other articles (17-25 years). The combined remission rate was 49% (95% CI: 15% to 83%) after excluding Rosenich alone and the remission rate for all studies was 40% (95% CI: 13% to 71%). The change in post-treatment HAMD scores from baseline after excluding Rosenich alone was (MD = -14.56, 95% CI: -22.22 to -6.90), and the combined HAMD scores for all studies was (MD = -12.78, 95% CI: -19.55 to -6.01). Furthermore, the exclusion of other individual studies similarly did not have a great impact on the pooled results, suggesting that the results of this meta-analysis were relatively reliable. Therefore, we did not exclude individual studies for re-analysis. The results of sensitivity analysis are shown in **Figures 7**–**9**.

#### 3.4.8 Publication bias

To ensure the validity of the meta-analysis results, we used funnel plot, Egger’s and Begg’s tests to identify publication bias of the main outcome measures. The results showed that the publication bias of the effect rate was not significant (P = 0.508), but the publication bias of the CRDs score was significant (P < 0.01). After we supplemented two articles with the trim and fill method, the results did not change, which further confirmed the reliability of our results.

## 4 Discussion

Our meta-analysis included 9 studies on the effect of NIBS in combination with antidepressants on depression in adolescents. No articles about children (6-12 years) were included in the analysis. The results showed that compared to baseline value, NIBS combined with antidepressants could decrease the depression score. The remission rate was 40% (95% CI: 13% to 71%). All adverse events incidence of 13% (95% CI: 5%, 23%). Furthermore, out of the 9 qualifying studies, 6 studies (77.8%) focused on patients with major depression. This suggests that the positive effects of using NIBS in combination with antidepressant medications may be more common in patients with major depression.

In research on depression, Qiu et al’s meta-analysis showed that rTMS can benefit children and adolescents with major depression in a relatively safe manner (48). Zheng et al. included three RCTs for a systematic review comparing the safety and efficacy of low-frequency rTMS versus sham stimulation in children and adolescents (mean age range from 14.5 to 17.5 years) with first-episode major depression, the results suggested that rTMS was beneficial and relatively safe for children and adolescents with major depression, but more high-quality RCTs are needed (34). In terms of the combined treatment of ECT, Pluijms et al. found that compared with the use of antidepressants alone, the combined use of antidepressants during ECT for major depression can enhance the efficacy (27). Elias et al. further found that ECT in combination with antidepressants significantly reduced the risk of relapse (49). The magnetic resonance imaging found that ECT did not cause brain damage, but rather induced a short-term increase in brain volume and modulated the effect by inducing changes in neuroplasticity (50, 51). The results of our meta-analysis showed that ECT combined with antidepressants was equally effective in adolescents, which was consistent with the results of the above-mentioned studies. In the context of TMS in combination with medication, results from previous meta-analysis showed that high-frequency rTMS accelerated clinical responses to antidepressants in patients with major depression and did not increase withdrawal rates (52). The neurophysiology mechanism may be related to cortical excitability, inhibitory imbalance, and the role of neuroplasticity (53). In this study, TMS combined with antidepressants significantly improved depression in adolescents, consistent with the findings in adults.

The pooled remission rate was 40%. Our findings were not entirely consistent with the results of a systematic review conducted by Zheng et al., in which two articles reported remission rates of 0% and 13.3% in the trial group. The differences in the results may be related to the number of included studies, the type of studies, the definition of outcomes and the choice of subjects. Three RCT studies were included in the systematic review by Zheng et al., and the results were qualitatively described. In addition, their review was conducted in children and adolescents diagnosed with first-episode major depression who did not receive any antidepressant therapy, and treatment remission was defined as a reduction in HAMD score by at least 75% (34). A previous meta-analysis incorporating 40 RCTs on psychological treatment for children and adolescents with depression revealed that among 38 different psychological treatments, the pooled remission rate and response rate in the psychological treatment group were 24% (95%CI: 19%-28%) and 41% (95% CI: 34%-48%). The results of subgroup analysis further showed that there was no significant difference in response rate between children and adolescents (54). A RCT conducted on young patients with bipolar depression revealed that the remission rates after 8 weeks of treatment were approximately 35.7% for the quetiapine group and 12.5% for the lurasidone group (55). Furthermore, an earlier study on the treatment of adolescent depression found that after 12 weeks of treatment, the complete remission rate was 37% for pharmacotherapy combined with cognitive therapy, 16% for cognitive-behavioral therapy, and 17% for placebo (19). When compared to current conventional treatments, NIBS as an adjunct to antidepressant medication appears to be a promising treatment option. Of note, in adults with major depression who received high-frequency TMS, the remission rate was approximately 18.6% (56). More than 50% of the adults who received ECT achieved remission (57). In summary, adolescents may benefit more than adults from NIBS combined with conventional medication for depression.

Previous neuroimaging studies showed that there were abnormal resting-state functional connections between brain regions in patients with major depression. Due to the disruption of network relationships, patients always showed some degree of medial prefrontal-medial parietal default mode network (DMN) dysfunction (58). Therefore, DMN is also a common target of TMS in the treatment of depression (59). Previous studies showed that TMS regulation may normalize the functional relationship between neural networks and improve clinical efficacy (60). It was reported that MDD patients had abnormal activities in the anterior cingulate cortex (ACC) and amygdala during emotional processing, often showing hyperactivation to negative stimuli and low activation to positive stimuli (61, 62). ECT may increase the activity of ACC in patients with major depression during negative emotion processing (62). Although we obtained very encouraging results, the subgroup analysis of the main outcome measure, CRDS, showed partial heterogeneity across and within subgroups. After subgroup analysis of symptom assessment scale, it was found that the different scales might be the potential source of heterogeneity. Compared with the uncorrected CDRS, the corrected CDRS-R could capture the change in depressive symptoms more effectively. The original CDRS was an observer-assessed tool used to measure the severity of depression in children and adolescents (63). The CDRS-R was a revised version of the CDRS, consisting of 17 semi-structured items, including cognitive, somatic, emotional, and psychomotor symptoms (64, 65). Shain et al. found that adolescents with major depression were able to show a faster improvement in CDRS-R scores (66). The reliability, validity and sensitivity to symptom changes of CDRS-R were also well verified (67). Subgroup analysis showed that there was no significant difference in the combined effects between different treatment times (2 weeks and 6-8 weeks). This may be due to small sample size effects or differences in subjects studied. In addition to patients with depression, some of the subjects in the depression group included by Shere et al. used insomnia medication. Wall et al. included adolescents with suicidal ideation at baseline, while Croarkin excluded individuals with mental disorders other than depression (43, 45, 47). A parallel double-blind sham controlled study of rTMS in the treatment of depression for 2-4 weeks found that rTMS had no adverse effect on neuropsychological function and was safe after 4 weeks of treatment (68). In addition, our subgroup analysis based on interventions found no differences in the efficacy of rTMS plus antidepressants versus TBS plus antidepressants. This could be explained by the fact that they were all subject to TMS. Although a few studies showed that TBS had similar or better efficacy in the treatment of depression compared with rTMS (69), this study did not identify differences, perhaps due to the number of studies and trial design.

In terms of safety, three studies reported safety events during treatment, with a pooled incidence of adverse events of approximately 13%. Adverse events included transient or mild head and neck pain, head irritation and muscle discomfort (32, 40, 45, 47). Previous meta-analysis of RCTs showed that it was a safe treatment with only some tolerable side effects, such as transient scalp discomfort or pain, which was similar to the results of this study (34, 70).

### 4.1 Limitations and Prospects

This meta-analysis highlighted the clinical safety and efficacy of NIBS in combination with antidepressants in adolescents with depression. Although our results provide some basis for future complementary therapies in the younger population with depression, there were still some limitations. First, the number of studies included in our analysis was small, 80% of which were single-arm trials, lacking a separate control group. This leads us to be cautious in interpreting this result, as we cannot identify confounding effects of antidepressants and psychological conditions. Second, owing to the lack of RCTs comparing NIBS with current conventional treatments (pharmacotherapy and psychotherapy), our results cannot support the replacement of traditional treatments with NIBS. RCT trials comparing the efficacy of NIBS and conventional treatments are warranted to further examine the true effects of NIBS. Third, in this meta-analysis, only two NIBS interventions were observed, of which 90% were TMS and 10% were ECT, which limited the generalization of our conclusions. Our results were more suitable for recommending TMS combined with antidepressants as a treatment for depression in adolescents. Fourth, owing to the majority of included studies being focused on patients with major depression, we can only draw conclusions about the benefits of NIBS for this type of patients. Fifth, although our investigation spanned both child and adolescent groups, in the end, no qualifying studies specifically focusing on children were incorporated, indicating that the available body of evidence pertains primarily to adolescent and young adult demographics. Sixth, there was significant heterogeneity in multiple outcomes after combination, which may be related to the diversity of study designs, small recruitment sizes, treatment parameters, and basic characteristics of the included subjects. The impact could not be further analyzed due to limitations in the number of studies with the same factors. Of the studies included in the analysis, the main focus was on TMS. As the optimal frequency, location, and intensity of stimulation in adolescents are unknown, further research is required to optimize the treatment. In addition, the age of the included population is somewhat controversial. There is currently no uniformity regarding the age boundaries of adolescents as a group, with two common criteria being 12-18 and 12-25 (which includes the youth group). We chose the latter criterion, which may have had a subtle effect on the results. Finally, as only one study reported on patient comorbidities (37), it is not clear whether there is some confounding regarding the role of comorbid psychiatric disorders in the treatment process.

In conclusion, NIBS combined with antidepressant therapy can significantly improve depression in adolescents with good safety and efficacy, especially rTMS stimulation scheme in the treatment of patients with major depression. However, due to the existence of high heterogeneity, careful interpretation is required.

## 5 Conclusions

Owing to the lack of studies in pediatric populations, the available evidence is more applicable to adolescent or young adult populations. In general, NIBS combined with antidepressants can effectively and safely improve the depressive symptoms of adolescents, especially rTMS for the treatment of depressive patients. In addition, there were a few minor adverse events, mainly pain at the stimulation site. Although our results support this therapeutic strategy, further multicenter studies with more rigorous designs, larger samples are still needed to verify these results.

## Data availability statement

The original contributions presented in the study are included in the article/**Supplementary Material**. Further inquiries can be directed to the corresponding author.

## Author contributions

YL: Conceptualization, Data curation, Formal Analysis, Methodology, Writing – original draft, Writing – review & editing. XL: Funding acquisition, Supervision, Writing – review & editing.
