# Supplementary material for: Efficacy and safety of non-invasive brain stimulation in combination with antidepressants in adolescents with depression: a systematic review and meta-analysis
Source: Front Psychiatry. 2024 Feb 15;15:1288338. doi: 10.3389/fpsyt.2024.1288338 (PMC10902042; doi:10.3389/fpsyt.2024.1288338)
Supplement: Supplementary file 1 [file DataSheet_1.docx]

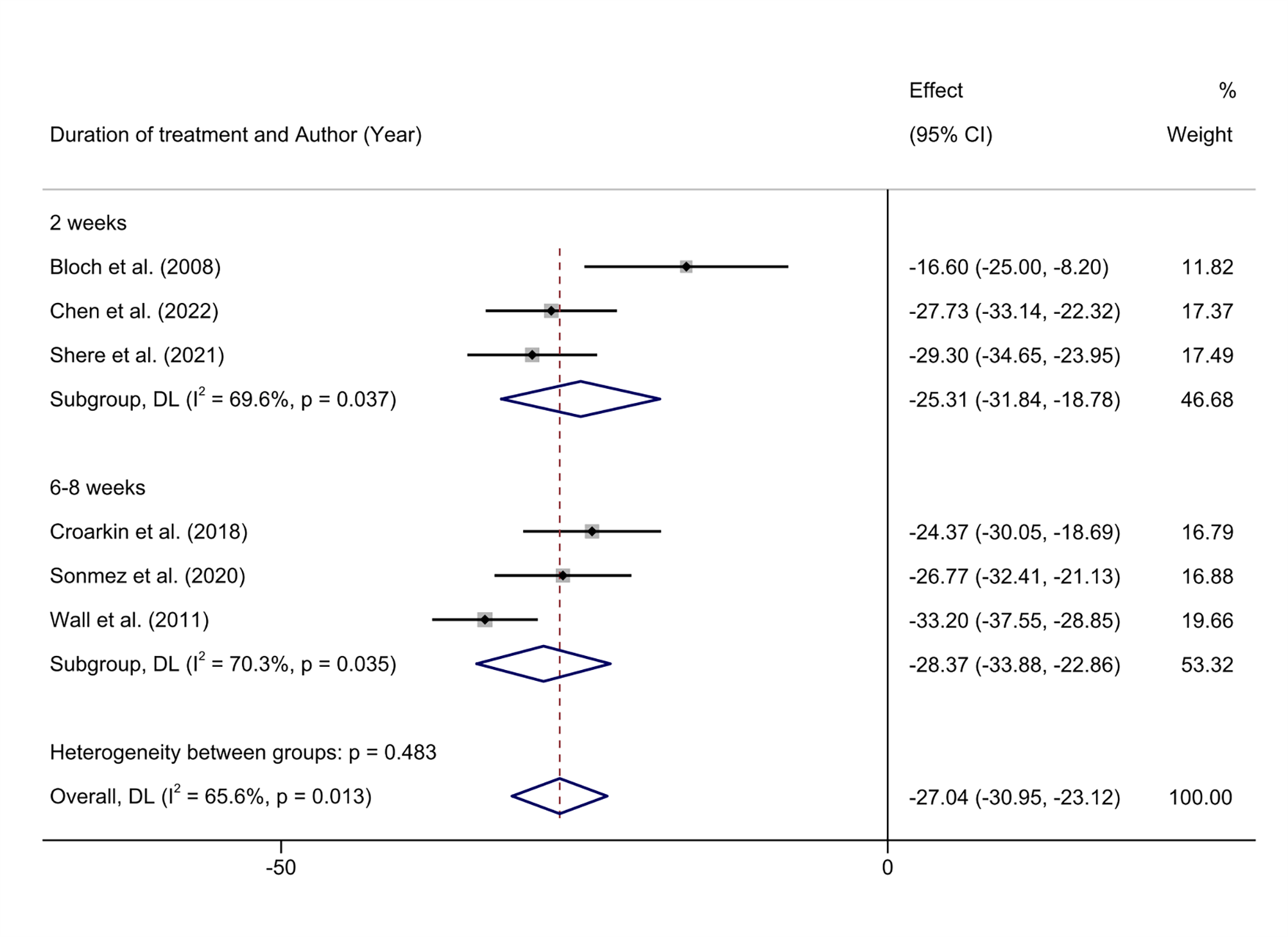


**Figure S1:** Subgroup analysis of CRDS scores based on treatment duration.


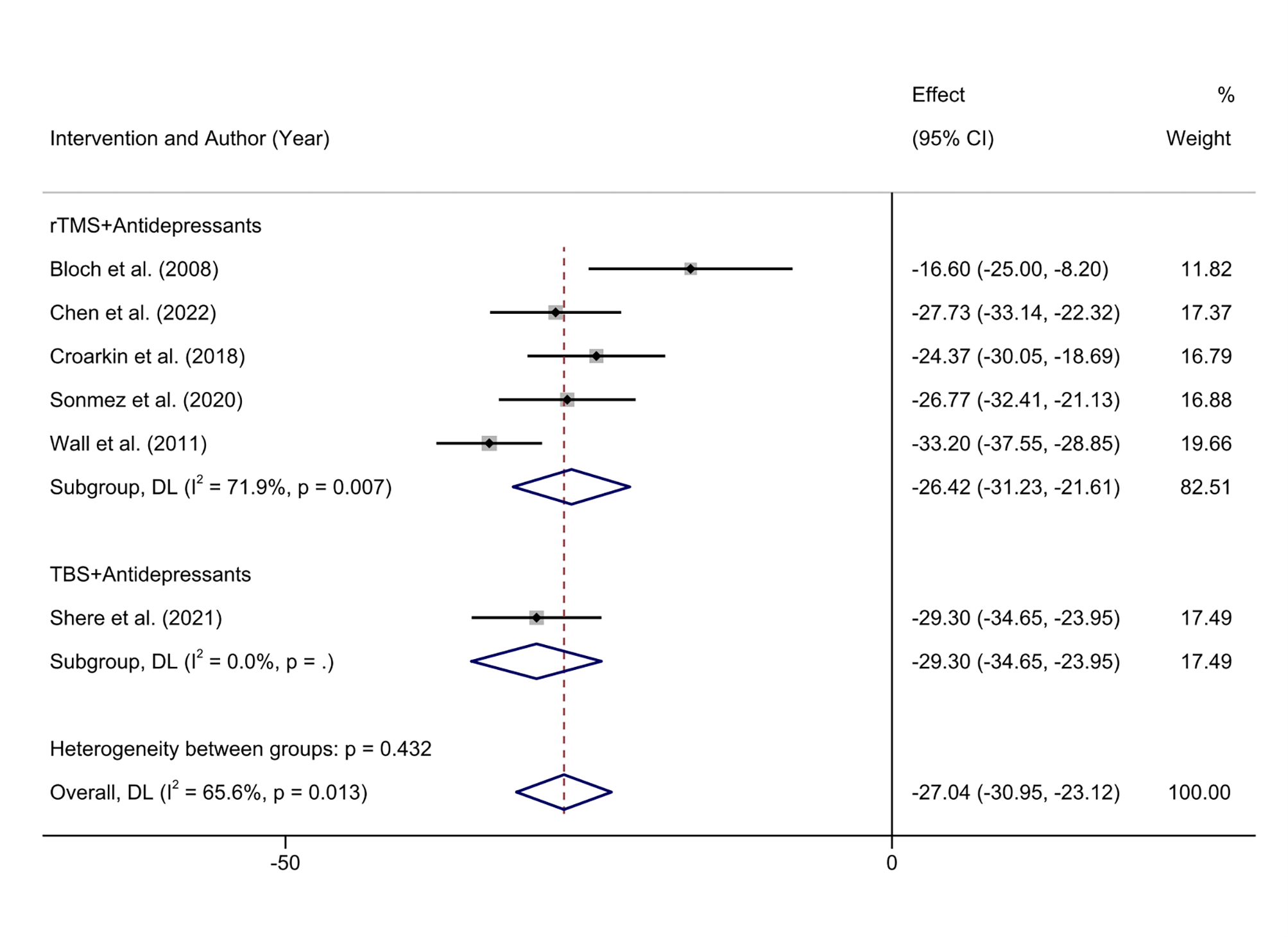


**Figure S2:** Subgroup analysis of CRDS scores based on intervention method.


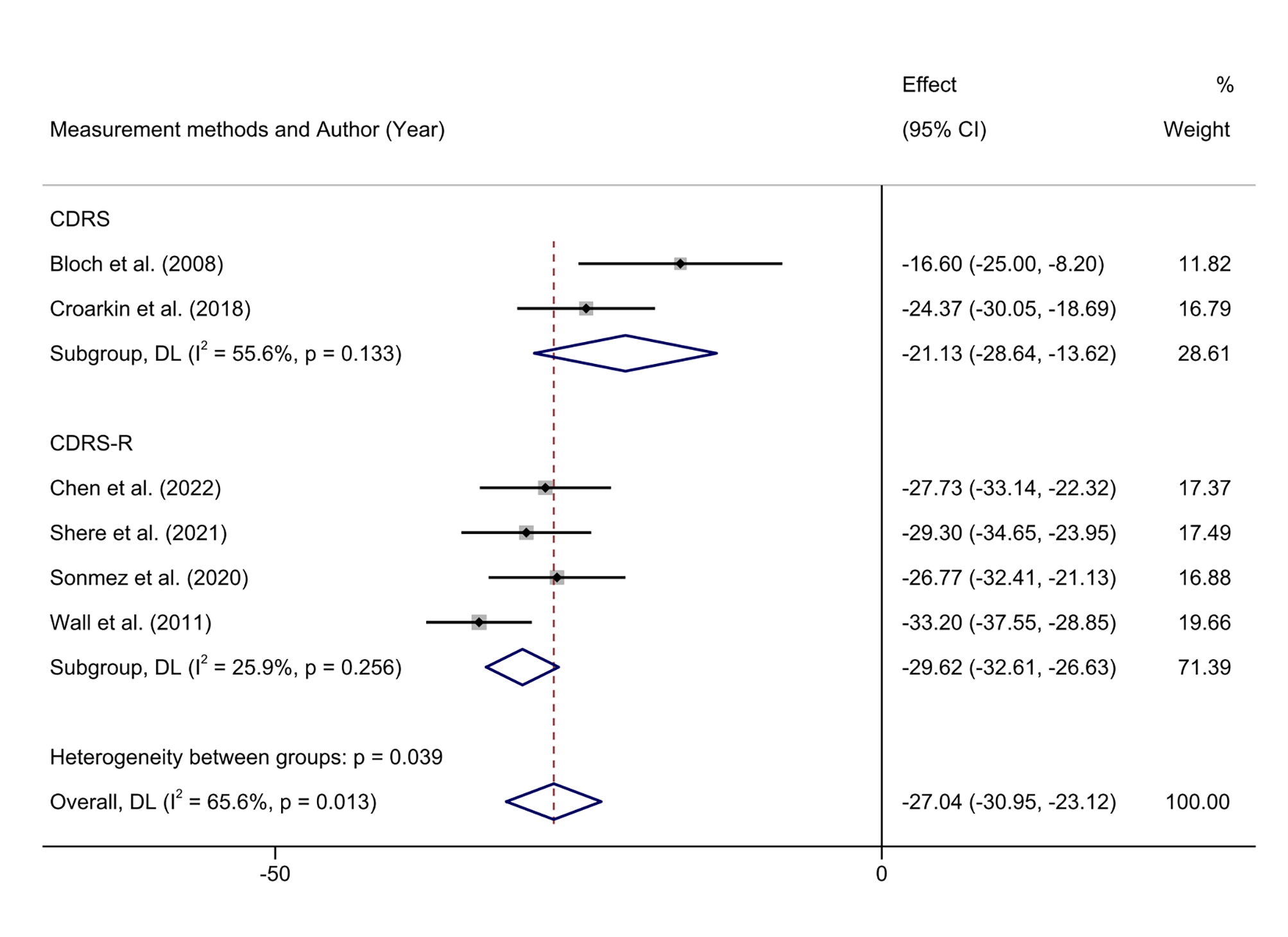


**Figure S3:** Subgroup analysis based on the CDRS scoring scale.


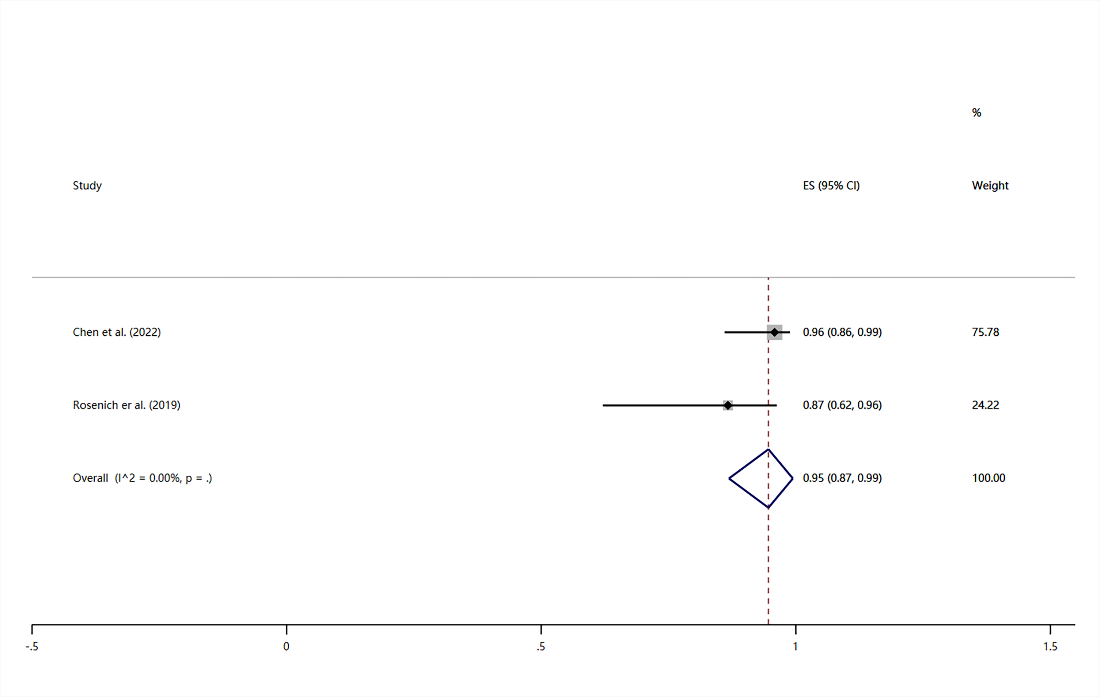


**Figure S4:** Forest plot of early improvement rate.


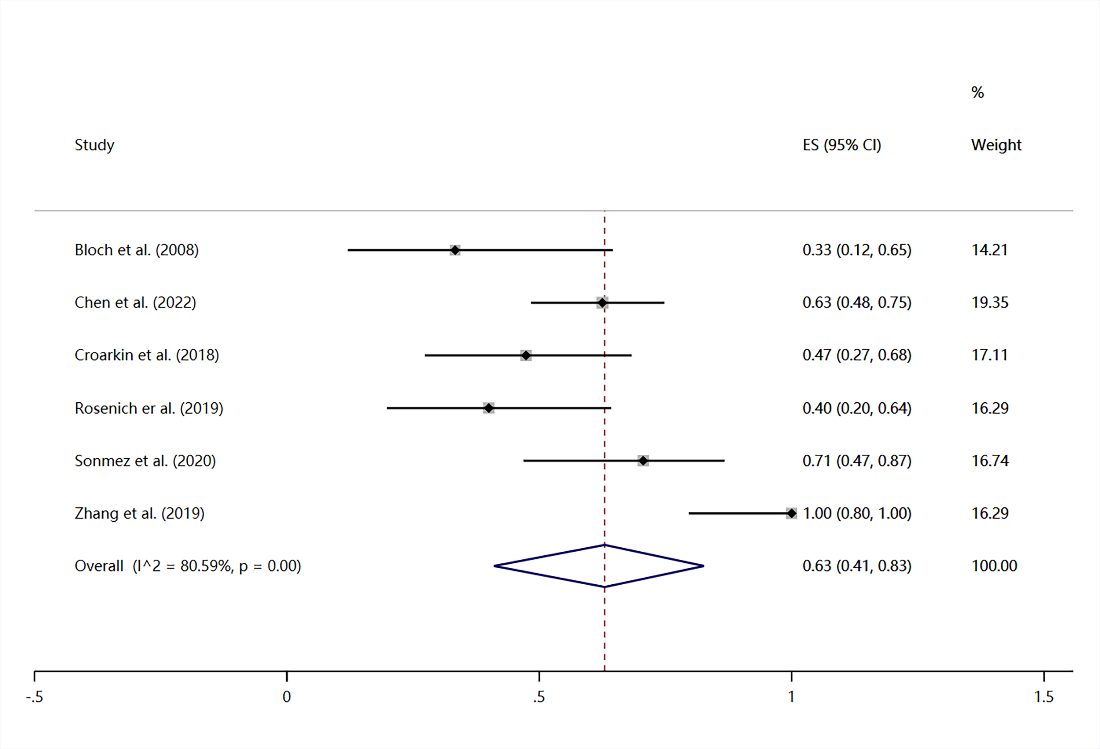


**Figure S5:** Forest plot of response rate.
