# Supplementary material for: Efficacy and safety of non-invasive brain stimulation in combination with antidepressants in adolescents with depression: a systematic review and meta-analysis
Source: Front Psychiatry. 2024 Feb 15;15:1288338. doi: 10.3389/fpsyt.2024.1288338 (PMC10902042; doi:10.3389/fpsyt.2024.1288338)
Supplement: Supplementary file 2 [file DataSheet_2.docx]

**Table S1:** Searching strategy

**1.Search strategy in Pubmed**

| #1 | "Depressive Disorder"[MeSH Terms] |
| --- | --- |
| #2 | "Adolescent"[MeSH Terms] |
| #3 | "Child"[MeSH Terms] |
| #4 | "Transcranial Magnetic Stimulation"[MeSH Terms] OR "Electroconvulsive Therapy"[MeSH Terms] OR "Magnetic Field Therapy"[MeSH Terms] OR "Transcranial Direct Current Stimulation"[MeSH Terms] |
| #5 | "Antidepressive Agents"[MeSH Terms] |
| #6 | ("Depressive Disorder"[Title/Abstract] OR "depression"[Title/Abstract] OR "Depressive Disorders"[Title/Abstract] OR "Depressive Neuroses"[Title/Abstract] OR "Depressive Neurosis"[Title/Abstract] OR "Endogenous Depression"[Title/Abstract] OR "Endogenous Depressions"[Title/Abstract] OR "Depressive Syndrome"[Title/Abstract] OR "Depressive Syndromes"[Title/Abstract] OR "Neurotic Depression"[Title/Abstract] OR "Neurotic Depressions"[Title/Abstract] OR "Melancholia"[Title/Abstract] OR "Melancholias"[Title/Abstract] OR "Unipolar Depression"[Title/Abstract] OR "Unipolar Depressions"[Title/Abstract] OR "central depression"[Title/Abstract] OR "clinical depression"[Title/Abstract] OR "depressive disease"[Title/Abstract] OR "depressive episode"[Title/Abstract] OR "depressive illness"[Title/Abstract] OR "depressive personality disorder"[Title/Abstract] OR "depressive state"[Title/Abstract] OR "depressive symptom"[Title/Abstract] OR "mental depression"[Title/Abstract] OR "parental depression"[Title/Abstract]) OR ("Depressive Disorder"[MeSH Terms]) |
| #7 | ("Child*"[Title/Abstract] OR "kid*"[Title/Abstract] OR "Adolescent"[Title/Abstract] OR "Adolescents"[Title/Abstract] OR "Adolescence"[Title/Abstract] OR "Teens"[Title/Abstract] OR "Teen"[Title/Abstract] OR "Teenagers"[Title/Abstract] OR "Teenager"[Title/Abstract] OR "Youth"[Title/Abstract] OR "Youths"[Title/Abstract]) OR ("Child"[MeSH Terms] OR "Adolescent"[MeSH Terms]) |
| #8 | ("Transcranial Magnetic Stimulation"[Title/Abstract] OR "Transcranial Magnetic Stimulations"[Title/Abstract] OR "rTMS"[Title/Abstract] OR "Transcranial Magnetic Stimulation, Repetitive"[Title/Abstract] OR "Stimulation, Transcranial Magnetic"[Title/Abstract] OR "Stimulations, Transcranial Magnetic"[Title/Abstract] OR "Magnetic Stimulation, Transcranial"[Title/Abstract] OR "Magnetic Stimulations, Transcranial"[Title/Abstract] OR "Electroconvulsive Therapy"[Title/Abstract] OR "Electroconvulsive Therapies"[Title/Abstract] OR "Electroshock Therapy"[Title/Abstract] OR "Electroshock Therapies"[Title/Abstract] OR "Electric Convulsive Therapies"[Title/Abstract] OR "Electric Convulsive Therapy"[Title/Abstract] OR "Electric Shock Therapies"[Title/Abstract] OR "Electric Shock Therapy"[Title/Abstract] OR "ECT"[Title/Abstract] OR "Magnetic Field Therapy"[Title/Abstract] OR "Magnetic Field Therapies"[Title/Abstract] OR "Electrically-Charged Magnetic Therapy"[Title/Abstract] OR "Electrically Charged Magnetic Therapy"[Title/Abstract] OR "Electrically-Charged Magnetic Therapies"[Title/Abstract] OR "Magnetic Stimulation Therapy"[Title/Abstract] OR "Magnetic Stimulation Therapies"[Title/Abstract] OR "Magnetotherapy"[Title/Abstract] OR "Static Magnetic Field Therapy"[Title/Abstract] OR "Electromagnetic Therapy"[Title/Abstract] OR "Electromagnetic Therapies"[Title/Abstract] OR "Transcranial Direct Current Stimulation"[Title/Abstract] OR "tDCS"[Title/Abstract] OR "Cathodal Stimulation tDCS"[Title/Abstract] OR "Cathodal Stimulation tDCSs"[Title/Abstract] OR "Transcranial Random Noise Stimulation"[Title/Abstract] OR "Transcranial Alternating Current Stimulation"[Title/Abstract] OR "Transcranial Electrical Stimulation"[Title/Abstract] OR "Transcranial Electrical Stimulations"[Title/Abstract] OR "Anodal Stimulation tDCS"[Title/Abstract] OR "Anodal Stimulation tDCSs"[Title/Abstract]) OR ("Transcranial Magnetic Stimulation"[MeSH Terms] OR "Electroconvulsive Therapy"[MeSH Terms] OR "Magnetic Field Therapy"[MeSH Terms] OR "Transcranial Direct Current Stimulation"[MeSH Terms]) |
| #9 | ("Antidepressive Agents"[Title/Abstract] OR "antidepressant"[Title/Abstract] OR "Antidepressive"[Title/Abstract] OR "western medicine"[Title/Abstract] OR "Antidepressants"[Title/Abstract] OR "Antidepressant"[Title/Abstract] OR "Thymoanaleptics"[Title/Abstract] OR "Thymoanaleptic"[Title/Abstract] OR "Thymoleptics"[Title/Abstract] OR "Thymoleptic"[Title/Abstract] OR "SSRI "[Title/Abstract] OR "SNRI"[Title/Abstract] OR "TCA"[Title/Abstract] OR "MAOI"[Title/Abstract] OR "NASSA"[Title/Abstract] OR "Bupropion"[Title/Abstract] OR "monoamine oxidase inhibitors"[Title/Abstract] OR "serotonin and noradrenaline reuptake inhibitors"[Title/Abstract] OR "serotonin uptake inhibitors"[Title/Abstract] OR "Anti-depressive"[Title/Abstract] OR "Anti-depression"[Title/Abstract] OR "Antidepression"[Title/Abstract]) OR ("Antidepressive Agents"[MeSH Terms]) |
| #10 | (((("Antidepressive Agents"[Title/Abstract] OR "antidepressant"[Title/Abstract] OR "Antidepressive"[Title/Abstract] OR "western medicine"[Title/Abstract] OR "Antidepressants"[Title/Abstract] OR "Antidepressant"[Title/Abstract] OR "Thymoanaleptics"[Title/Abstract] OR "Thymoanaleptic"[Title/Abstract] OR "Thymoleptics"[Title/Abstract] OR "Thymoleptic"[Title/Abstract] OR "SSRI "[Title/Abstract] OR "SNRI"[Title/Abstract] OR "TCA"[Title/Abstract] OR "MAOI"[Title/Abstract] OR "NASSA"[Title/Abstract] OR "Bupropion"[Title/Abstract] OR "monoamine oxidase inhibitors"[Title/Abstract] OR "serotonin and noradrenaline reuptake inhibitors"[Title/Abstract] OR "serotonin uptake inhibitors"[Title/Abstract] OR "Anti-depressive"[Title/Abstract] OR "Anti-depression"[Title/Abstract] OR "Antidepression"[Title/Abstract]) OR ("Antidepressive Agents"[MeSH Terms])) AND (("Transcranial Magnetic Stimulation"[Title/Abstract] OR "Transcranial Magnetic Stimulations"[Title/Abstract] OR "rTMS"[Title/Abstract] OR "Transcranial Magnetic Stimulation, Repetitive"[Title/Abstract] OR "Stimulation, Transcranial Magnetic"[Title/Abstract] OR "Stimulations, Transcranial Magnetic"[Title/Abstract] OR "Magnetic Stimulation, Transcranial"[Title/Abstract] OR "Magnetic Stimulations, Transcranial"[Title/Abstract] OR "Electroconvulsive Therapy"[Title/Abstract] OR "Electroconvulsive Therapies"[Title/Abstract] OR "Electroshock Therapy"[Title/Abstract] OR "Electroshock Therapies"[Title/Abstract] OR "Electric Convulsive Therapies"[Title/Abstract] OR "Electric Convulsive Therapy"[Title/Abstract] OR "Electric Shock Therapies"[Title/Abstract] OR "Electric Shock Therapy"[Title/Abstract] OR "ECT"[Title/Abstract] OR "Magnetic Field Therapy"[Title/Abstract] OR "Magnetic Field Therapies"[Title/Abstract] OR "Electrically-Charged Magnetic Therapy"[Title/Abstract] OR "Electrically Charged Magnetic Therapy"[Title/Abstract] OR "Electrically-Charged Magnetic Therapies"[Title/Abstract] OR "Magnetic Stimulation Therapy"[Title/Abstract] OR "Magnetic Stimulation Therapies"[Title/Abstract] OR "Magnetotherapy"[Title/Abstract] OR "Static Magnetic Field Therapy"[Title/Abstract] OR "Electromagnetic Therapy"[Title/Abstract] OR "Electromagnetic Therapies"[Title/Abstract] OR "Transcranial Direct Current Stimulation"[Title/Abstract] OR "tDCS"[Title/Abstract] OR "Cathodal Stimulation tDCS"[Title/Abstract] OR "Cathodal Stimulation tDCSs"[Title/Abstract] OR "Transcranial Random Noise Stimulation"[Title/Abstract] OR "Transcranial Alternating Current Stimulation"[Title/Abstract] OR "Transcranial Electrical Stimulation"[Title/Abstract] OR "Transcranial Electrical Stimulations"[Title/Abstract] OR "Anodal Stimulation tDCS"[Title/Abstract] OR "Anodal Stimulation tDCSs"[Title/Abstract]) OR ("Transcranial Magnetic Stimulation"[MeSH Terms] OR "Electroconvulsive Therapy"[MeSH Terms] OR "Magnetic Field Therapy"[MeSH Terms] OR "Transcranial Direct Current Stimulation"[MeSH Terms]))) AND (("Child*"[Title/Abstract] OR "kid*"[Title/Abstract] OR "Adolescent"[Title/Abstract] OR "Adolescents"[Title/Abstract] OR "Adolescence"[Title/Abstract] OR "Teens"[Title/Abstract] OR "Teen"[Title/Abstract] OR "Teenagers"[Title/Abstract] OR "Teenager"[Title/Abstract] OR "Youth"[Title/Abstract] OR "Youths"[Title/Abstract]) OR ("Child"[MeSH Terms] OR "Adolescent"[MeSH Terms]))) AND (("Depressive Disorder"[Title/Abstract] OR "depression"[Title/Abstract] OR "Depressive Disorders"[Title/Abstract] OR "Depressive Neuroses"[Title/Abstract] OR "Depressive Neurosis"[Title/Abstract] OR "Endogenous Depression"[Title/Abstract] OR "Endogenous Depressions"[Title/Abstract] OR "Depressive Syndrome"[Title/Abstract] OR "Depressive Syndromes"[Title/Abstract] OR "Neurotic Depression"[Title/Abstract] OR "Neurotic Depressions"[Title/Abstract] OR "Melancholia"[Title/Abstract] OR "Melancholias"[Title/Abstract] OR "Unipolar Depression"[Title/Abstract] OR "Unipolar Depressions"[Title/Abstract] OR "central depression"[Title/Abstract] OR "clinical depression"[Title/Abstract] OR "depressive disease"[Title/Abstract] OR "depressive episode"[Title/Abstract] OR "depressive illness"[Title/Abstract] OR "depressive personality disorder"[Title/Abstract] OR "depressive state"[Title/Abstract] OR "depressive symptom"[Title/Abstract] OR "mental depression"[Title/Abstract] OR "parental depression"[Title/Abstract]) OR ("Depressive Disorder"[MeSH Terms])) |

**2.Search strategy in Cochrane**

| #1 | MeSH descriptor: [Depressive Disorder] explode all trees |
| --- | --- |
| #2 | (Depressive Disorder):ti,ab,kw OR (depression):ti,ab,kw OR (Depressive Disorders):ti,ab,kw OR (Depressive Neuroses):ti,ab,kw OR (Depressive Neurosis):ti,ab,kw OR (Endogenous Depression):ti,ab,kw OR (Endogenous Depressions):ti,ab,kw OR (Depressive Syndrome):ti,ab,kw OR (Depressive Syndromes):ti,ab,kw OR (Neurotic Depression):ti,ab,kw OR (Neurotic Depressions):ti,ab,kw OR (Melancholia):ti,ab,kw OR (Melancholias):ti,ab,kw OR (Unipolar Depression):ti,ab,kw OR (Unipolar Depressions):ti,ab,kw OR (central depression):ti,ab,kw OR (clinical depression):ti,ab,kw OR (depressive disease):ti,ab,kw OR (depressive episode):ti,ab,kw OR (depressive illness):ti,ab,kw OR (depressive personality disorder):ti,ab,kw OR (depressive state):ti,ab,kw OR (depressive symptom):ti,ab,kw OR (mental depression):ti,ab,kw OR (parental depression):ti,ab,kw |
| #3 | MeSH descriptor: [Adolescent] explode all trees |
| #4 | MeSH descriptor: [Child] explode all trees |
| #5 | (Child*):ti,ab,kw OR (kid*):ti,ab,kw OR (Adolescent):ti,ab,kw OR (Adolescents):ti,ab,kw OR (Adolescence):ti,ab,kw OR (Teens):ti,ab,kw OR (Teen):ti,ab,kw OR (Teenagers):ti,ab,kw OR (Teenager):ti,ab,kw OR (Youth):ti,ab,kw OR (Youths):ti,ab,kw |
| #6 | #1 or #2 |
| #7 | #3 or #4 or #5 |
| #8 | #6 and #7 |
| #9 | MeSH descriptor: [Transcranial Magnetic Stimulation] explode all trees |
| #10 | (Transcranial Magnetic Stimulation):ti,ab,kw OR (Transcranial Magnetic Stimulations):ti,ab,kw OR (rTMS):ti,ab,kw OR (Transcranial Magnetic Stimulation, Repetitive):ti,ab,kw OR (Stimulation, Transcranial Magnetic):ti,ab,kw OR (Stimulations, Transcranial Magnetic):ti,ab,kw OR (Magnetic Stimulation, Transcranial):ti,ab,kw OR (Magnetic Stimulations, Transcranial):ti,ab,kw |
| #11 | #9 or #10 |
| #12 | MeSH descriptor: [Electroconvulsive Therapy] explode all trees |
| #13 | (Electroconvulsive Therapy):ti,ab,kw OR (Electroconvulsive Therapies):ti,ab,kw OR (Electroshock Therapy):ti,ab,kw OR (Electroshock Therapies):ti,ab,kw OR (Electric Convulsive Therapies):ti,ab,kw OR (Electric Convulsive Therapy):ti,ab,kw OR (Electric Shock Therapies):ti,ab,kw OR (Electric Shock Therapy):ti,ab,kw OR (ECT):ti,ab,kw |
| #14 | #12 or #13 |
| #15 | MeSH descriptor: [Magnetic Field Therapy] explode all trees |
| #16 | (Magnetic Field Therapy):ti,ab,kw OR (Magnetic Field Therapies):ti,ab,kw OR (Electrically-Charged Magnetic Therapy):ti,ab,kw OR (Electrically Charged Magnetic Therapy):ti,ab,kw OR (Electrically-Charged Magnetic Therapies):ti,ab,kw OR (Magnetic Stimulation Therapy):ti,ab,kw OR (Magnetic Stimulation Therapies):ti,ab,kw OR (Magnetotherapy):ti,ab,kw OR (Static Magnetic Field Therapy):ti,ab,kw OR (Electromagnetic Therapy):ti,ab,kw OR (Electromagnetic Therapies):ti,ab,kw |
| #17 | #15 or #16 |
| #18 | MeSH descriptor: [Transcranial Direct Current Stimulation] explode all trees |
| #19 | (Transcranial Direct Current Stimulation):ti,ab,kw OR (tDCS):ti,ab,kw OR (Cathodal Stimulation tDCS):ti,ab,kw OR (Cathodal Stimulation tDCSs):ti,ab,kw OR (Transcranial Random Noise Stimulation):ti,ab,kw OR (Transcranial Alternating Current Stimulation):ti,ab,kw OR (Transcranial Electrical Stimulation):ti,ab,kw OR (Transcranial Electrical Stimulations):ti,ab,kw OR (Anodal Stimulation tDCS):ti,ab,kw OR (Anodal Stimulation tDCSs):ti,ab,kw |
| #20 | #18 or #19 |
| #21 | #11 or #14 or #17 or #120 |
| #22 | #8 and #20 |
| #23 | MeSH descriptor: [Antidepressive Agents] explode all trees |
| #24 | (Antidepressive Agents):ti,ab,kw OR (antidepressant):ti,ab,kw OR (Antidepressive):ti,ab,kw OR (western medicine):ti,ab,kw OR (Antidepressants):ti,ab,kw OR (Antidepressant):ti,ab,kw OR (Thymoanaleptics):ti,ab,kw OR (Thymoanaleptic):ti,ab,kw OR (Thymoleptics):ti,ab,kw OR (Thymoleptic):ti,ab,kw OR (SSRI ):ti,ab,kw OR (SNRI):ti,ab,kw OR (TCA):ti,ab,kw OR (MAOI):ti,ab,kw OR (NASSA):ti,ab,kw OR (Bupropion):ti,ab,kw OR (monoamine oxidase inhibitors):ti,ab,kw OR (serotonin and noradrenaline reuptake inhibitors):ti,ab,kw OR (serotonin uptake inhibitors):ti,ab,kw OR (Anti-depressive):ti,ab,kw OR (Anti-depression):ti,ab,kw OR (Antidepression):ti,ab,kw |
| #25 | #23 or #24 |
| #26 | #22 and #25 |

**3.Search strategy in Embase**

| #1 | 'depression'/exp |
| --- | --- |
| #2 | 'adolescent'/exp |
| #3 | 'child'/exp |
| #4 | 'transcranial magnetic stimulation'/exp |
| #5 | 'electroconvulsive therapy'/exp |
| #6 | 'transcranial direct current stimulation'/exp |
| #7 | 'antidepressant agent'/exp |
| #8 | 'depressive disorder':ab,ti OR 'depression':ab,ti OR 'depressive disorders':ab,ti OR 'depressive neuroses':ab,ti OR 'depressive neurosis':ab,ti OR 'endogenous depression':ab,ti OR 'endogenous depressions':ab,ti OR 'depressive syndrome':ab,ti OR 'depressive syndromes':ab,ti OR 'neurotic depression':ab,ti OR 'neurotic depressions':ab,ti OR 'melancholia':ab,ti OR 'melancholias':ab,ti OR 'unipolar depression':ab,ti OR 'unipolar depressions':ab,ti OR 'central depression':ab,ti OR 'clinical depression':ab,ti OR 'depressive disease':ab,ti OR 'depressive episode':ab,ti OR 'depressive illness':ab,ti OR 'depressive personality disorder':ab,ti OR 'depressive state':ab,ti OR 'depressive symptom':ab,ti OR 'mental depression':ab,ti OR 'parental depression':ab,ti |
| #9 | 'child*':ab,ti OR 'kid*':ab,ti OR 'adolescent':ab,ti OR 'adolescents':ab,ti OR 'adolescence':ab,ti OR 'teens':ab,ti OR 'teen':ab,ti OR 'teenagers':ab,ti OR 'teenager':ab,ti OR 'youth':ab,ti OR 'youths':ab,ti |
| #10 | transcranial magnetic stimulation':ab,ti OR 'transcranial magnetic stimulations':ab,ti OR 'rtms':ab,ti OR 'transcranial magnetic stimulation, repetitive':ab,ti OR 'stimulation, transcranial magnetic':ab,ti OR 'stimulations, transcranial magnetic':ab,ti OR 'magnetic stimulation, transcranial':ab,ti OR 'magnetic stimulations, transcranial':ab,ti OR 'electroconvulsive therapy':ab,ti OR 'electroconvulsive therapies':ab,ti OR 'electroshock therapy':ab,ti OR 'electroshock therapies':ab,ti OR 'electric convulsive therapies':ab,ti OR 'electric convulsive therapy':ab,ti OR 'electric shock therapies':ab,ti OR 'electric shock therapy':ab,ti OR 'ect':ab,ti OR 'magnetic field therapy':ab,ti OR 'magnetic-field therapies':ab,ti OR 'electrically-charged-magnetic- therapy':ab,ti OR 'electrically charged-magnetic-therapy':ab,ti OR 'electrically-charged-magnetic-therapies':ab,ti OR 'magnetic-stimulation-therapy':ab,ti OR 'magnetic-stimulation therapies':ab,ti OR 'magnetotherapy':ab,ti OR 'static-magnetic-field-therapy':ab,ti OR 'electromagnetic-therapy':ab,ti OR 'electromagnetic therapies':ab,ti OR 'transcranial direct current stimulation':ab,ti OR 'tdcs':ab,ti OR 'cathodal stimulation-tdcs':ab,ti OR 'cathodal stimulation tdcss':ab,ti OR 'transcranial random noise stimulation':ab,ti OR 'transcranial alternating current stimulation':ab,ti OR 'transcranial electrical stimulation':ab,ti OR 'transcranial electrical stimulations':ab,ti OR 'anodal stimulation-tdcs':ab,ti OR 'anodal stimulation tdcss':ab,ti |
| #11 | 'antidepressive agents':ab,ti OR 'antidepressive':ab,ti OR 'western medicine':ab,ti OR 'antidepressants':ab,ti OR 'antidepressant':ab,ti OR 'thymoanaleptics':ab,ti OR 'thymoanaleptic':ab,ti OR 'thymoleptics':ab,ti OR 'thymoleptic':ab,ti OR 'ssri':ab,ti OR 'snri':ab,ti OR 'tca':ab,ti OR 'maoi':ab,ti OR 'nassa':ab,ti OR 'bupropion':ab,ti OR 'monoamine oxidase inhibitors':ab,ti OR 'serotonin and noradrenaline reuptake inhibitors':ab,ti OR 'serotonin uptake inhibitors':ab,ti OR 'anti-depressive':ab,ti OR 'anti-depression':ab,ti OR 'antidepression':ab,ti 150604 |
| #12 | #1 OR #8 |
| #13 | #2 OR #3 OR #9 |
| #14 | #4 OR #5 OR #6 OR #10 |
| #15 | #7 OR #11 |
| #16 | #12 AND #13 AND #14 AND #15 |

**4.Search strategy in Web of Science**

| #1 | TS=(Depressive Disorder) OR TS=(depression) OR TS=(Depressive Disorders) OR TS=(Depressive Neuroses) OR TS=(Depressive Neurosis) OR TS=(Endogenous Depression) OR TS=(Endogenous Depressions) OR TS=(Depressive Syndrome) OR TS=(Depressive Syndromes) OR TS=(Neurotic Depression) OR TS=(Neurotic Depressions) ORTS=(Melancholia) OR TS=(Melancholias) OR TS=(Unipolar Depression) OR TS=(Unipolar Depressions) OR TS=(central depression) OR TS=(clinical depression) OR TS=(depressive disease) OR TS=(depressive episode) OR TS=(depressive illness) OR TS=(depressive personality disorder) OR TS=(depressive state) OR TS=(depressive symptom) OR TS=(mental depression) OR TS=(parental depression) |
| --- | --- |
| #2 | TS=(child*) OR TS=(kid*) OR TS=(Adolescent) OR TS=(Adolescents) OR TS=(Adolescence) OR TS=(Teens) OR TS=(Teen) OR TS=(Teenagers) OR TS=(Teenager) OR TS=(Youth) OR TS=(Youths) |
| #3 | TS=(Transcranial Magnetic Stimulation) OR TS=(Transcranial Magnetic Stimulations) OR TS=(rTMS) OR TS=(Transcranial Magnetic Stimulation, Repetitive) OR TS=(Stimulation, Transcranial Magnetic) OR TS=(Stimulations, Transcranial Magnetic) OR TS=(Magnetic Stimulation, Transcranial) OR TS=(Magnetic Stimulations, Transcranial) OR TS=(Electroconvulsive Therapy) OR TS=(Electroconvulsive Therapies) OR TS=(Electroshock Therapy) OR TS=(Electroshock Therapies) OR TS=(Electric Convulsive Therapies) OR TS=(Electric Convulsive Therapy) OR TS=(Electric Shock Therapies) OR TS=(Electric Shock Therapy) OR TS=(ECT) OR TS=(Magnetic Field Therapy) OR TS=(Magnetic Field Therapies) OR TS=(Electrically-Charged Magnetic Therapy) OR TS=(Electrically Charged Magnetic Therapy) OR TS=(Electrically-Charged Magnetic Therapies) OR TS=(Magnetic Stimulation Therapy) OR TS=(Magnetic Stimulation Therapies) OR TS=(Magnetotherapy) OR TS=(Static Magnetic Field Therapy) OR TS=(Electromagnetic Therapy) OR TS=(Electromagnetic Therapies) OR TS=(Transcranial Direct Current Stimulation) OR TS=(tDCS) OR TS=(Cathodal Stimulation tDCS) OR TS=(Cathodal Stimulation tDCSs) OR TS=(Transcranial Random Noise Stimulation) OR TS=(Transcranial Alternating Current Stimulation) OR TS=(Transcranial Electrical Stimulation) OR TS=(Transcranial Electrical Stimulations) OR TS=(Anodal Stimulation tDCS) OR TS=(Anodal Stimulation tDCSs) |
| #4 | "TS=(Antidepressive Agents) OR TS=(antidepressant) OR TS=(Antidepressive) OR TS=(western medicine) OR TS=(Antidepressants) OR TS=(Antidepressant) OR TS=(Thymoanaleptics) OR TS=(Thymoanaleptic) OR TS=(Thymoleptics) OR TS=(Thymoleptic) OR TS=(SSRI ) OR TS=(SNRI) OR TS=(TCA) OR TS=(MAOI) OR TS=(NASSA) OR TS=(Bupropion) OR TS=(monoamine oxidase inhibitors) OR TS=(serotonin and noradrenaline reuptake inhibitors) OR TS=(serotonin uptake inhibitors) OR TS=(Anti-depressive) OR TS=(Anti-depression) OR TS=(Antidepression) |
| #5 | "#4 AND #3 AND #2 AND #1 |
